# 1,4,7-Triazacyclononane-Based Chelators for the Complexation of [^186^Re]Re- and [^99m^Tc]Tc-Tricarbonyl Cores

**DOI:** 10.1021/acs.inorgchem.3c01934

**Published:** 2023-09-08

**Authors:** Rebecca Hoerres, Heather M. Hennkens

**Affiliations:** † Department of Chemistry, University of Missouri, Columbia, Missouri 65211, United States; ‡ Research Reactor Center, University of Missouri, Columbia, Missouri 65211, United States

## Abstract

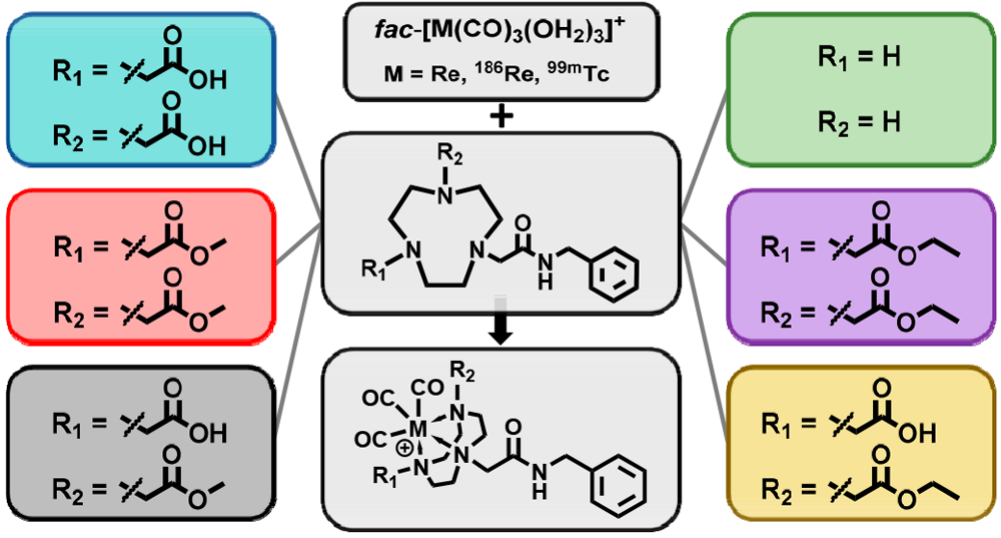

Metal complexes with the general formula [M^I^(CO)_3_(k^3^-L)]^+^, where M = Re, ^186^Re, or ^99m^Tc and L = 1,4,7-triazacyclononane (TACN), NOTA, or NODAGA chelators, have previously been conjugated to peptide-based biological targeting vectors and investigated as potential theranostic radiopharmaceuticals. The promising results demonstrated by these bioconjugate complexes prompted our exploration of other TACN-based chelators for suitability for (radio)labeling with the [M(CO)_3_]^+^ core. In this work, we investigated the role of the TACN pendant arms in complexation of the [M(CO)_3_]^+^ core through (radio)labeling of TACN chelators bearing acid, ester, mixed acid–ester, or no pendant functional groups. The chelators were synthesized from TACN, characterized, and (radio)labeled with nonradioactive Re-, [^186^Re]Re-, and [^99m^Tc]Tc-tricarbonyl cores. The nonfunctionalized (**3**), diacid (**4**), and monoacid monoester (**7** and **8**) chelators underwent direct labeling, while the diester (**M-5** and **M-6**) complexes required indirect synthesis from **M-4**. All six chelators demonstrated stable radiometal coordination. The ester-bearing derivatives, which exhibited more lipophilic character than their acid-bearing counterparts, were prone to ester hydrolysis over time, making them less suitable for radiopharmaceutical development. These studies confirmed that the TACN pendant functional groups were key to efficient labeling with the [M(CO)_3_]^+^ core, with ionizable pendant arms favored over nonionizable pendant arms.

## Abstract 2

Six 1,4,7-triazacyclononane-based chelators bearing acid, ester, mixed acid−ester, or no pendant functional groups on the ring nitrogen atoms were successfully synthesized, characterized, and labeled with the [M(CO)_3_]^+^ core (M = Re, ^186^Re, ^99m^Tc). Stable metal chelation was observed for all resulting complexes. The number of ionizable pendant arms correlated with labeling yields, supporting the hypothesis that electrostatic attraction between the negatively charged pendant functional groups and the positively charged metal tricarbonyl core aids labeling.

## 1 Introduction

Technetium-99m and rhenium-186/188 are of great interest in the development of radiopharmaceuticals due to their favorable decay characteristics. Tc-99m (*t*_1/2_ = 6 h) is a diagnostic imaging radionuclide useful for single-photon emission-computed tomography via the detection of its 140 keV (89%) γ-ray. Re-186 (*t*_1/2_ = 3.7 days) and ^188^Re (*t*_1/2_ = 17 h) can be used as therapeutic radionuclides because they both decay by β^–^ emission (maximum β^–^ energies of 1.08 and 2.11 MeV, respectively). Technetium and rhenium are congeners, resulting in the similar chemical and physical properties that these metals share that form the basis of their potential to be used as a theranostic radionuclide matched pair.

Both technetium and rhenium can exist in oxidation states ranging from 1– to 7+, with the most common oxidation states used for radiopharmaceutical development being 1+ and 5+.^1^ As radiotracer reagents, they exist in aqueous solution as the permetallate form, MO_4_^–^ (7+ oxidation state). The metals can be reduced and stabilized in the 1+ oxidation state through the formation of a tricarbonyltriaqua complex, [M^I^(CO)_3_(OH_2_)_3_]^+^. Procedures for synthesis of the [M^I^(CO)_3_(OH_2_)_3_]^+^ precursors (M = Re, ^186^Re, ^188^Re, and ^99m^Tc) are well established in the literature.^2−4^ The labile water ligands in these precursors can be replaced by the reaction with a tridentate chelating agent, two ligands for 2 + 1 chelation, or three monodentate ligands to form metal tricarbonyl complexes.^5−7^

Chelators bearing nitrogen donor atoms or combinations of nitrogen, oxygen, and/or sulfur donor atoms are commonly reacted with the [M^I^(CO)_3_(OH_2_)_3_]^+^ precursor to form [M^I^(CO)_3_L] complexes. The cyclic 1,4,7-triazacyclononane (TACN)-based chelators NOTA and NODAGA are of particular interest for the [M(CO)_3_]^+^ cores because they bear pendant acid arms that increase the overall hydrophilicity of the metal complexes.^8^ Various reports exist on derivatives of TACN-based chelators (radio)labeled with gallium, copper, zinc, iron, and manganese,^7,9,10^ with relatively few reports on the use of derivatized TACN chelators with the [M(CO)_3_]^+^ cores. In one example, structural studies with Re- and ^99g^Tc-labeled NOTA complexes demonstrated that the metal center is coordinated via the three TACN nitrogen atoms on the opposing face of the metal tricarbonyl core, with no coordination between the metal and the pendant acid arms.^11^ In other examples, [^99m^Tc]Tc- and [^186^Re]Re-tricarbonyl-labeled NOTA and NODAGA complexes showed excellent stability when conjugated to several biological targeting vectors.^12−14^

These previous studies demonstrated that the pharmacokinetic profiles of the M(CO)_3_-labeled NOTA and NODAGA bioconjugates were heavily influenced by the hydrophilicity and charge of the overall complexes. For [^99m^Tc][Tc^I^(CO)_3_L] somatostatin receptor targeting bioconjugates bearing the sst_2_-ANT targeting peptide, the NOTA derivative outperformed its NODAGA counterpart in a mouse tumor model with significantly higher tumor uptake at 1 h postinjection.^14^ The overall charge for the [^99m^Tc][Tc^I^(CO)_3_(NOTA-sst_2_-ANT)] compound was neutral, while the [^99m^Tc][Tc^I^(CO)_3_(NODAGA-sst_2_-ANT)] compound had an overall charge of 1–. The increased hydrophilicity of the NODAGA bioconjugate led to fast renal excretion of the metal complex, resulting in lower tumor uptake. The NOTA bioconjugate was excreted more slowly, also predominantly via the renal pathway, leading to greater bioavailability and higher tumor uptake. Conversely, for M^I^(CO)_3_-labeled NOTA/NODAGA bioconjugates bearing gastrin-releasing peptide receptor targeting peptide RM2, the NODAGA derivative outperformed its NOTA counterpart with higher tumor uptake and retention through 24 h.^12^ In this case, the overall charges of the NOTA and NODAGA compounds were 1– and 2–, respectively. The NODAGA complex again showed increased hydrophilicity, resulting in fast and predominantly renal clearance, while the NOTA complex showed mixed clearance that was dominated by the hepatobiliary route. These studies demonstrated that a small change in the bifunctional chelator can have a profound impact on the pharmacokinetic profile of the bioconjugate.

These early results prompted our exploration of other TACN-based chelators that may also be suitable for (radio)labeling with the [M(CO)_3_]^+^ cores. We chose to explore TACN chelators bearing pendant ester arms because replacing the acid functional groups with esters was a relatively small structural change expected to have a large impact on the hydrophilicity and charge of the resulting metal tricarbonyl complexes. To evaluate how modifying the pendant functional groups on the TACN backbone affects the (radio)labeling yields, stability, and hydrophilicity of M(CO)_3_-labeled complexes, we present herein the synthesis and evaluation of six different TACN-based tridentate ligands, each with one of its TACN nitrogen atoms functionalized with an *N*-benzylacetamide arm to serve as a small-molecule surrogate for a targeting vector. The modified TACN chelators additionally bear either no other pendant arms on the TACN backbone nitrogen atoms (overall 1+ charged metal complexes at physiological pH) or those with acid (1– charged), ester (1+ charged), or a combination of acid–ester (neutral) functional groups.

## 2 Materials and Methods

### 2.1 General Procedures

All chemicals were of reagent-grade and were purchased from Sigma-Aldrich (St. Louis, MO) and Fisher Scientific (Pittsburgh, PA) unless otherwise stated. The 1,4,7-triazacyclononane (TACN) chelator was purchased from CheMatech (Dijon, France). High-performance liquid chromatography (HPLC) solvents were of HPLC-grade, were filtered through membrane or nylon filters, and were degassed prior to use. HPLC analyses and purifications were performed on Shimadzu Nexera or Shimadzu Prominence HPLC systems with photodiode array detectors connected inline to NaI(Tl) detectors. HPLC chromatograms were analyzed at the 210 nm wavelength for all TACN-based chelators and at 254 nm for all Re-labeled compounds. For HPLC and radio-HPLC analyses, a Thermo Fisher Scientific BetaBasic C18 column (150 mm × 4.6 mm, 5 μm) was used with a flow rate of 1 mL/min. For semipreparative HPLC purification, a Phenomenex Luna C18 column (250 × 10 mm, 10 μm) was used with a flow rate of 4 mL/min. HPLC analyses and purifications were run under a binary linear gradient with pumps A and B containing water (with 0.1% trifluoroacetic acid (TFA)) and methanol (with 0.1% TFA), respectively. The following gradients were used: 5–95% B in A over 18 min (Method 1), 15–65% B in A over 10 min (Method 2), 30–95% B in A over 25 min (Method 3), and 50–95% B in A over 14 min (Method 4).

High-resolution electrospray ionization mass spectrometry (HRMS) analyses were performed on an LTQ Orbitrap XL mass spectrometer. All mass spectrometry data were analyzed by using Xcalibur *Qual Browser*, version 2.2 (Thermo Fisher Scientific). IR spectra were recorded on a Thermo Scientific Nicolet Summit Pro Fourier transform infrared spectrometer. Wavelengths between 500 and 4000 cm^–1^ were recorded. ^1^H and ^13^C NMR spectra were acquired on a Bruker Avance III 500 or 600 MHz spectrometer. The NMR data were analyzed with Bruker *TopSpin*, version 4.0.9. Elemental analyses were performed by combustion analysis at Atlantic Microlab (Norcross, GA).

Na[^99m^Tc]TcO_4_ was eluted from a ^99^Mo/^99m^Tc generator (Curium, St. Louis, MO) donated by Mid-America Isotopes Inc. (Ashland, MO). Na[^186^Re]ReO_4_ was produced at the University of Missouri Research Reactor by neutron irradiation of Al(^185^ReO_4_)_3_ targets, and the specific activity at the end of irradiation was 56–74 GBq/mg (1.5–2.0 Ci/mg). ***Caution!** Both ^99m^Tc and ^186^Re are radioactive nuclides and emit β^–^ particles (^186^Re) and/or γ-rays (^99m^Tc and ^186^Re) upon decay. All handling of these radionuclides was conducted by appropriately trained personnel within laboratories approved for radioactive material use and under proper radiation safety procedures with appropriate protective shielding.*

Activity measurements for samples containing ^99m^Tc and ^186^Re were made on a Capintec CRC-55tR dose calibrator (Ramsey, NJ), an ORTEC 4890 NaI(Tl) well detector, or an ORTEC HPGe GEM20-70 high-purity germanium detector (Oak Ridge, TN) coupled to a Canberra DSA-LX multichannel analyzer (Meriden, CT). The HPGe data were analyzed using Canberra *Genie 2000* (3.3) software. Radio thin-layer chromatography (radio-TLC) analyses were developed on A81-24 saturation pads (Analtech, Newark, DE) with saline (Developer 1) or 50% acetonitrile in saline (Developer 2) as the mobile phase and analyzed on an Eckert and Ziegler AR-2000 radio-TLC imager scanner (Hopkinton, MA).

### 2.2 Synthesis of *N*-Benzyl-2-(1,4,7-triazonan-1-yl)acetamide (TACN-benzylamide, **3**)

**Scheme 1:**
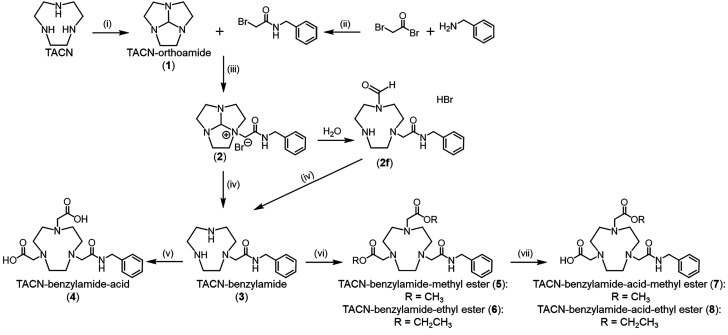
Synthesis of TACN-Based Chelators Conditions: (i) *N*,*N*-dimethylformamide dimethyl acetal (1 equiv), acetonitrile, 80 °C, 93%; (ii) dichloromethane, 0 °C to RT, 76%; (iii) tetrahydrofuran, RT, 61%; (iv) NaOH (3.1 equiv), water, 90 °C, 85%; (v) chloroacetic acid (2 equiv), NaOH (4 equiv), water, 60 °C, 60%; (vi) methyl/ethyl bromoacetate (2 equiv), K_2_CO_3_ (2 equiv), acetonitrile, RT, 46% (R = methyl), 54% (R = ethyl); (vii) R–OH/H_2_O (1:1), NaOH (0.1-0.2 equiv), RT, 27% (R = methyl), 32% (R = ethyl).

The synthesis of the TACN-based chelators is shown in Scheme 1. Compounds **1** and **2** and *N*-benzyl-2-bromoacetamide were synthesized following a literature procedure.^11^ Details for the synthesis of these compounds can be found in the Supporting Information. The synthesis of chelator **3** was adapted from the literature.^11^ Briefly, compound **2** (200 mg, 0.70 mmol) was dissolved in 5 mL of water with sodium hydroxide (NaOH; 87 mg, 2.2 mmol), and the reaction was heated at 90 °C in an oil bath. The reaction progress was monitored by analytical HPLC (Method 1, *t*_R_ = 7.2 min). Subsequent amounts of NaOH (30 mg, 0.75 mmol) were added every 6 h until all of compound **2** had reacted. The water was removed under reduced pressure, and the product was HPLC-purified using semipreparative HPLC (Method 2, *t*_R_ = 8.3 min) in 5–10 mg batches. The HPLC eluate was removed under reduced pressure to yield **3** as a yellow oil. Isolated yield: 85% (155 mg). The product was characterized by HRMS and ^1^H and ^13^C NMR. HRMS. Calcd for C_15_H_24_N_4_O ([M + H]^+^): *m*/*z* 277.2023. Found: *m*/*z* 277.2000. ^1^H NMR (D_2_O, 600 MHz): δ_H_ 7.32–7.34 (m, 2H), 7.24–7.28 (m, 3H), 4.34 (s, 2H), 3.59 (s, 4H), 3.52 (s, 2H), 3.22 (t, 4H, *J* = 5.6 Hz), 2.98 (t, 4H, *J* = 5.6 Hz). ^13^C NMR (D_2_O, 600 MHz): δ_C_ 173.6, 137.6, 128.8, 127.6, 127.3, 56.2, 48.9, 44.1, 43.2, 42.8. The NMR spectra match the previously reported spectra.^11^

### 2.3 Synthesis of TACN-benzylamide Acid (**4**)

Chelator **3** (28 mg, 0.10 mmol) and chloroacetic acid (19 mg, 0.20 mmol) were dissolved in 2 mL of water and NaOH (16 mg, 0.40 mmol). The reaction was heated at 60 °C for 4 h. The reaction progress was monitored by analytical HPLC (Method 1, *t*_R_ = 9.2 min), and the crude product was purified by semipreparative HPLC (Method 1, *t*_R_ = 11.4 min). Isolated yield: 60% (24 mg). The product, a clear oil, was characterized by HRMS and ^1^H and ^13^C NMR. HRMS. Calcd for C_19_H_28_N_4_O_5_ ([M + H]^+^): *m*/*z* 393.2133. Found: *m*/*z* 393.2093. ^1^H NMR ((CD_3_)_2_SO, 600 MHz): δ_H_ 8.73 (t, 1H, *J* = 5.3 Hz), 7.33–7.35 (m, 2H) 7.26–7.29 (m, 3H), 4.34 (d, 2H, *J* = 5.3 Hz), 3.80 (s, 2H), 3.62 (s, 4H), 3.01 (m, 12H). ^13^C NMR (CD_3_CN, 600 MHz): δ_C_ 172.0, 167.4, 139.2, 128.8, 127.8, 127.4, 56.9, 55.1, 50.9, 49.6, 48.6, 42.7.

### 2.4 Synthesis of TACN-benzylamide Methyl Ester (**5**)

Chelators **5** and **6** were synthesized according to an adapted literature procedure.^15^ Chelator **3** (30 mg, 0.11 mmol) was dissolved in 6.5 mL of dry acetonitrile. Potassium carbonate (33 mg, 0.24 mmol) and methyl bromoacetate (37 mg, 0.24 mmol) were added, and the reaction mixture was stirred at room temperature (RT) for 18 h. The reaction progress was monitored by HPLC (Method 1, *t*_R_ = 12.1 min). Upon reaction completion, potassium carbonate was filtered off, and the final product was purified by semipreparative HPLC (Method 2, *t*_R_ = 9.0 min) in 5–10 mg batches. Isolated yield: 46% (21 mg). The product, a clear oil, was characterized by HRMS and ^1^H and ^13^C NMR. HRMS. Calcd for C_21_H_32_N_4_O_5_ ([M + H]^+^): *m*/*z* 421.2446. Found: *m*/*z* 421.2416. ^1^H NMR (CDCl_3_, 600 MHz): δ_H_ 8.95 (s, 1H), 7.30–7.33 (m, 4H), 7.23–7.25 (m, 1H), 4.44 (d, 2H), 4.21 (s, 2H), 3.74 (s, 6H), 3.56 (s, 4H), 3.31 (m, 4H), 2.99 (m, 4H), 2.83 (m, 4H). ^13^C NMR (CDCl_3_, 600 MHz): δ_C_ 171.3, 165.0, 137.7, 128.6, 127.7, 127.4, 57.2, 55.2, 52.5, 51.9, 50.2, 47.1, 43.6.

### 2.5 Synthesis of TACN-benzylamide Ethyl Ester (**6**)

Chelator **6** was synthesized in the same manner as chelator **5**, except for the use of ethyl bromoacetate instead of methyl bromoacetate. The final product was HPLC purified (Method 2, *t*_R_ = 10.3 min) to yield **6** as a light-yellow oil. Isolated yield: 54% (26 mg). The product was characterized by HRMS and ^1^H and ^13^C NMR. HRMS. Calcd for C_23_H_36_N_4_O_5_ ([M + H]^+^): *m*/*z* 449.2759. Found: *m*/*z* 449.2730. ^1^H NMR (CDCl_3_, 600 MHz): δ_H_ 8.28 (s, 1H), 7.31–7.26 (m, 5H), 4.43 (d, 2H), 4.16 (q, 4H, *J* = 6.9 Hz), 4.07 (s, 2H), 3.57 (s, 4H), 3.24 (s, 4H), 3.06 (s, 4H), 2.91 (s, 4H), 1.67 (t, 6H, *J* = 6.9 Hz). ^13^C NMR (CDCl_3_, 600 MHz): δ_C_ 170.7, 165.6, 137.6, 128.6, 127.7, 127.4, 61.3, 57.5, 55.2, 52.1, 50.1, 47.5, 43.5, 14.1.

### 2.6 Synthesis of TACN-benzylamide Acid Methyl Ester (**7**)

Chelator **7** was synthesized by hydrolyzing one pendant ester arm on chelator **5**. Chelator **5** (62 mg, 0.15 mmol) was dissolved in 50% methanol in water (2 mL), and NaOH (1 mg, 0.025 mmol) was added. The reaction mixture was stirred at RT for 3 days. Every 24 h, the reaction progress was monitored by HPLC (Method 1, *t*_R_ = 10.9 min), and NaOH (1 mg, 0.025 mmol) was added as needed. The reaction was intentionally not completed to avoid hydrolysis of both esters. Once at least 50% of the starting material, **5**, had been hydrolyzed, the product was purified by semipreparative HPLC (Method 3, *t*_R_ = 11.1 min). Isolated yield: 27% (16 mg). The product was characterized by HRMS and ^1^H and ^13^C NMR. HRMS. Calcd for C_20_H_30_N_4_O_5_ ([M + H]^+^): *m*/*z* 407.2289. Found: *m*/*z* 407.2246. ^1^H NMR (MeOD, 600 MHz): δ_H_ 7.32–7.36 (m, 4H), 7.25–7.30 (m, 1H), 4.43 (s, 2H), 3.82 (s, 2H), 3.79 (s, 2H), 3.70 (s, 3H), 3.68 (s, 2H), 3.06–3.21 (m, 12H). ^13^C NMR (MeOD, 600 MHz): δ_C_ 172.1, 171.8, 168.8, 139.1, 128.8, 127.8, 127.5, 56.5, 55.0, 54.9, 51.8, 51.5, 51.0, 50.0, 49.1, 48.0, 47.5, 42.7.

### 2.7 Synthesis of TACN-benzylamide Acid Ethyl Ester (**8**)

Chelator **8** was synthesized in a manner similar to that of chelator **7**, except for using 50% ethanol in water as the solvent. The product was purified by semipreparative HPLC (Method 3, *t*_R_ = 12.4 min). Isolated yield: 32% (23 mg). The product was characterized by HRMS and ^1^H and ^13^C NMR. HRMS. Calcd for C_21_H_32_N_4_O_5_ ([M + H]^+^): *m*/*z* 421.2446. Found: *m*/*z* 421.2407. ^1^H NMR ((CD_3_)_2_SO), 600 MHz): δ_H_ 8.80 (t, 1H, *J* = 5.8 Hz), 7.29–7.34 (m, 5H), 4.35 (d, 2H, *J* = 5.8 Hz), 4.10 (q, 2H, *J* = 7.1 Hz), 3.88 (s, 2H), 3.65 (s, 2H), 3.61 (s, 2H), 3.02 (m, 12H), 1.21 (t, 3H, *J* = 7.1 Hz). ^13^C NMR ((CD_3_)_2_SO), 600 MHz): δ_C_ 172.0, 171.2, 166.8, 139.1, 128.8, 127.8, 127.5, 60.6, 56.5, 55.03, 55.00, 51.5, 51.1, 50.0, 49.1, 48.1, 47.6, 42.7, 14.6.

### 2.8 Preparation of the [Re(CO)_3_(OH_2_)_3_](NO_3_) Precursor

(NEt_4_)_2_[Re(CO)_3_Br_3_] was synthesized according to a literature procedure.^4^ Details for the synthesis can be found in the Supporting Information. The (NEt_4_)_2_[Re(CO)_3_Br_3_] solid was stored in a desiccator and converted as needed to the [Re(CO)_3_(OH_2_)_3_](NO_3_) precursor for labeling studies. (NEt_4_)_2_[Re(CO)_3_Br_3_] (40 mg, 0.052 mmol) was dissolved in 1.5 mL of water, and silver nitrate (26.5 mg, 0.16 mmol) in 500 μL of water was added, which resulted in the immediate formation of an off-white precipitate. The reaction was stirred in a thermomixer at RT for 30 min. The precipitate was filtered off, and the [Re(CO)_3_(OH_2_)_3_](NO_3_) product was analyzed by HPLC (Method 1, *t*_R_ = 4.4 min).

### 2.9 Synthesis of *fac*-[Re(CO)_3_(TACN-benzylamide)]^+^ (**Re-3**)

**Scheme 2:**
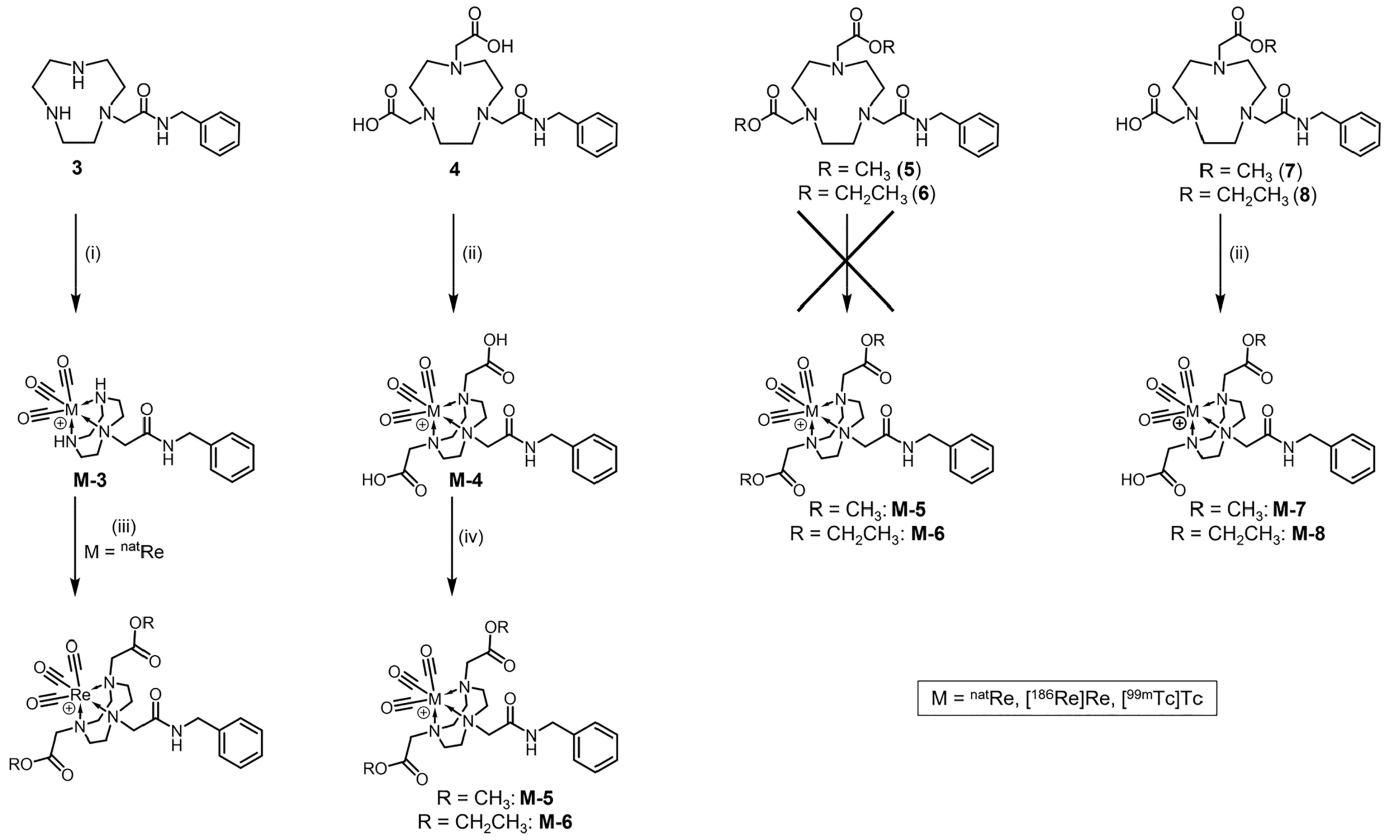
Synthesis of the M(CO)_3_-Labeled Chelators Conditions: (i) [M(CO)_3_(OH_2_)_3_]^+^, PBS buffer (pH 7), 95 °C; (ii) [M(CO)_3_(OH_2_)_3_]^+^, MES buffer (pH 5), 95 °C; (iii) methyl/ethyl bromoacetate (40 equiv), cesium carbonate (20 equiv), acetonitrile, RT; (iv) methanol/ethanol, thionyl chloride, 60 °C.

The synthesis of the metal complexes is shown in Scheme 2. Chelator **3** (9 mg, 0.033 mmol) in 90 μL of water was combined with [Re(CO)_3_(OH_2_)_3_](NO_3_) (30 mg, 0.039 mmol) in 600 μL of water, and the reaction mixture was diluted to 5 mL with phosphate-buffered saline (PBS; 1 mM, pH 7). The reaction was heated at 95 °C for 3 h in a thermomixer. The reaction progress was monitored by HPLC (Method 1, *t*_R_ = 12.9 min), and the product was purified by semipreparative HPLC (Method 1, *t*_R_ = 15.1 min). Isolated yield: 35% (6.3 mg). The product, a white powder, was characterized by HRMS, IR, ^1^H and ^13^C NMR, and elemental analysis. HRMS. Calcd for C_18_H_24_N_4_O_4_^185^Re^+^ ([M]^+^): *m*/*z* 545.1322. Found: *m*/*z* 545.1309, which matches the theoretical isotope distribution. IR (solid, cm^–1^): 3200, 2025, 1890, 1658, 1129. ^1^H NMR (CD_3_CN, 600 MHz): δ_H_ 7.36–7.38 (m, 2H), 7.29–7.33 (m, 3H), 7.21 (s, 1H), 5.72 (s, 2H), 4.39 (d, 2H), 4.20 (s, 2H), 3.41–3.48 (m, 4H), 3.33–3.38 (m, 2H), 3.15–3.20 (m, 2H), 2.98–3.04 (m, 2H), 2.91–2.95 (m, 2H). ^13^C NMR (CD_3_CN, 600 MHz): δ_C_ 195.4, 167.6, 138.5, 128.5, 127.5, 127.2, 65.8, 56.5, 51.3, 49.9, 42.5. Elem anal. Calcd for C_18_H_24_N_4_O_4_Re^+^(TFA)_2_(H_2_O): C, 33.34; H, 3.56; N, 7.07. Found: C, 33.27; H, 3.31; N, 6.97.

### 2.10 Synthesis of *fac*-[Re(CO)_3_(TACN-benzylamide acid)]^+^ (**Re-4**)

Chelator **4** (12 mg, 0.031 mmol) in 15 μL of dimethyl sulfoxide was combined with [Re(CO)_3_(OH_2_)_3_](NO_3_) (35 mg, 0.046 mmol) in 1 mL of water, and the reaction was diluted to a total volume of 4 mL with 2-(*N*-morpholino)ethanesulfonic acid (MES) buffer (0.2 M, pH 5). The reaction was heated at 95 °C for 5 h in a thermomixer. The reaction progress was monitored by HPLC (Method 1, *t*_R_ = 14.1 min), and the product was purified by semipreparative HPLC (Method 4, *t*_R_ = 7.3 min). Isolated yield: 57% (12 mg). The product, a white powder, was characterized by HRMS, IR, ^1^H and ^13^C NMR, and elemental analysis. HRMS. Calcd for C_22_H_28_N_4_O_8_^185^Re^+^ ([M]^+^): *m*/*z* 661.1431. Found: *m*/*z* 661.1428, which matches the theoretical isotope distribution. IR (solid, cm^–1^): 2400–3000, 2032, 1901, 1727, 1647, 1177, 1135. ^1^H NMR (CD_3_CN, 600 MHz): δ_H_ 7.36–7.39 (m, 2H), 7.29–7.33 (m, 3H), 7.26 (t, 1H, *J* = 5.9 Hz), 4.40 (d, 2H, *J* = 5.9 Hz), 4.34 (s, 4H), 4.24 (s, 2H), 3.63–3.70 (m, 8H), 3.49–3.53 (m, 4H). ^13^C NMR (CD_3_CN, 600 MHz): δ_C_ 194.3, 169.4, 167.4, 138.4, 128.5, 127.5, 127.3, 66.1, 64.9, 57.6, 57.3, 42.6. Elem anal. Calcd for C_22_H_28_N_4_O_8_Re^+^(TFA)_2_: C, 35.06; H, 3.39; N, 6.29. Found: C, 35.19; H, 3.46; N, 6.37.

### 2.11 Synthesis of *fac*-[Re(CO)_3_(TACN-benzylamide methyl ester)]^+^ (**Re-5**)

All attempts at direct labeling of chelators **5** and **6** with [Re(CO)_3_(OH_2_)_3_](NO_3_) were unsuccessful. Therefore, an alternative synthetic route was used (Scheme 2).

**Re-4** (25 mg, 0.038 mmol) was dissolved in 2 mL of dry methanol in a glass vial, and the vial was sealed with a crimp cap. The reaction was heated to 60 °C in an oil bath. A Pasteur pipet was inserted into the septum to vent the reaction, and thionyl chloride (600 μL, 8.2 mmol) was added dropwise over 2 min. The reaction continued heating at 60 °C for 1 h. The solvent was removed under reduced pressure, and the product was purified by semipreparative HPLC (Method 4, *t*_R_ = 8.4 min). Isolated yield: 78% (20 mg). The product, a white powder, was characterized by HRMS, IR, ^1^H and ^13^C NMR, and elemental analysis. HRMS. Calcd for C_24_H_32_N_4_O_8_^185^Re^+^ ([M]^+^): *m*/*z* 689.1744. Found: *m*/*z* 689.1690, which matches the theoretical isotope distribution. IR (solid, cm^–1^): 2032, 1900, 1737, 1665, 1130. ^1^H NMR ((CD_3_)_2_CO, 500 MHz): δ_H_ 8.21 (s, 1H), 7.32–7.34 (m, 4H), 7.26–7.30 (m, 1H), 4.58 (s, 4H), 4.52 (s, 2H), 4.47 (d, 2H), 3.93–3.97 (m, 8H), 3.78–3.82* (m, 4H), 3.78* (s, 6H) (* indicates overlapping peaks). ^13^C NMR ((CD_3_)_2_CO, 600 MHz): δ_C_ 194.2, 168.7, 167.3, 138.6, 128.4, 127.7, 127.2, 66.4, 65.0, 58.1, 58.0, 57.9, 51.8, 42.6. Elem anal. Calcd for C_24_H_32_N_4_O_8_Re^+^(TFA)_2_: C, 36.60; H, 3.73; N, 6.10. Found: C, 36.60; H, 3.76; N, 6.01.

### 2.12 Synthesis of *fac*-[Re(CO)_3_(TACN-benzylamide ethyl ester)]^+^ (**Re-6**)

**Re-6** was synthesized from **Re-4** following the same procedure as that used to synthesize **Re-5**, except dry ethanol was used as the solvent. Isolated yield: 58% (16 mg). The product, a white powder, was characterized by HRMS, IR, ^1^H and ^13^C NMR, and elemental analysis. HRMS. Calcd for C_26_H_36_N_4_O_8_^185^Re^+^ ([M]^+^): *m*/*z* 717.2057. Found: *m*/*z* 717.2001, which matches the theoretical isotope distribution. IR (solid, cm^–1^): 2032, 1900, 1732, 1662, 1194. ^1^H NMR ((CD_3_)_2_CO, 500 MHz): δ_H_ 8.56 (s, 1H), 7.31–7.36 (m, 4H), 7.26–7.28 (m, 1H), 4.56 (s, 4H), 4.51 (s, 2H), 4.46 (d, 2H), 4.25 (q, 4H, *J* = 7.1 Hz), 3.91–3.98 (m, 8H), 3.80–3.85 (m, 4H), 1.28 (t, 6H, *J* = 7.1 Hz). ^13^C NMR ((CD_3_)_2_CO, 600 MHz): δ_C_ 194.2, 168.3, 167.2, 138.7, 128.4, 127.7, 127.1, 66.5, 65.2, 61.3, 58.1, 57.93, 57.90, 42.5, 13.4. Elem anal. Calcd for C_26_H_36_N_4_O_8_Re^+^(TFA)_2_: C, 38.06; H, 4.05; N, 5.92. Found: C, 38.34; H, 4.06; N, 5.89.

### 2.13 Synthesis of *fac*-[Re(CO)_3_(TACN-benzylamide acid methyl ester)]^+^ (**Re-7**)

Chelator **7** (19 mg, 0.047 mmol) in 15 μL of dimethyl sulfoxide was combined with [Re(CO)_3_(OH_2_)_3_](NO_3_) (54 mg, 0.070 mmol) in 1 mL of water, and the reaction was diluted to a total volume of 4 mL of MES (0.2 M, pH 5). The reaction was heated at 95 °C for 5 h in a thermomixer. The reaction progress was monitored by analytical HPLC (Method 1, *t*_R_ = 14.6 min), and the product was purified by semipreparative HPLC (Method 4, *t*_R_ = 7.7 min). Isolated yield: 58% (19 mg). The product, a white powder, was characterized by HRMS, IR, ^1^H and ^13^C NMR, and elemental analysis. HRMS. Calcd for C_23_H_30_N_4_O_8_^185^Re^+^ ([M]^+^) *m*/*z* 675.1588. Found: *m*/*z* 675.1578, which matches the theoretical isotope distribution. IR (solid, cm^–1^): 2400–3000, 2032, 1900, 1734, 1654, 1157, 1132. ^1^H NMR (CD_3_CN, 600 MHz): δ_H_ 7.37–7.39 (m, 2H), 7.29–7.33 (m, 3H), 7.25 (t, 2H, *J* = 5.9 Hz), 4.40 (d, 2H, *J* = 5.9 Hz), 4.35 (s, 2H), 4.32 (s, 2H), 4.24 (s, 2H), 3.77 (s, 3H), 3.62–3.73 (m, 8H), 3.48–3.55 (m, 4H). ^13^C NMR (CD_3_CN, 600 MHz): δ_C_ 194.2, 169.4, 168.8, 167.4, 138.4, 128.5, 127.5, 127.3, 66.1, 64.9, 64.7, 57.7, 57.6, 57.5, 57.2, 52.1, 42.4. Elem anal. Calcd for C_23_H_30_N_4_O_8_Re^+^(TFA)_2_: C, 35.84; H, 3.57; N, 6.19. Found: C, 35.83; H, 3.57; N, 6.26.

### 2.14 Synthesis of *fac*-[Re(CO)_3_(TACN-benzylamide acid ethyl ester)]^+^ (**Re-8**)

Chelator **8** (12 mg, 0.029 mmol) in 50 μL of water was combined with [Re(CO)_3_(OH_2_)_3_](NO_3_) (44 mg, 0.057 mmol) in 0.5 mL of water, and the reaction was diluted to a total volume of 3 mL of MES (0.2 M, pH 5). The reaction was heated at 95 °C for 5 h in a thermomixer. The reaction progress was monitored by analytical HPLC (Method 1, *t*_R_ = 15.8 min), and the product was purified by semipreparative HPLC (Method 4, *t*_R_ = 8.8 min). Isolated yield: 34% (6 mg). The product, a white powder, was characterized by HRMS, IR, ^1^H and ^13^C NMR, and elemental analysis. HRMS. Calcd for C_24_H_32_N_4_O_8_^185^Re^+^ ([M]^+^): *m*/*z* 689.1745. Found: *m*/*z* 689.1679. IR (solid, cm^–1^): 2400–3000, 2033, 1903, 1730, 1658, 1179, 1131. ^1^H NMR ((CD_3_)_2_CO, 600 MHz): δ_H_ 8.26 (s, 1H), 7.28–7.35 (m, 5H), 4.55–4.58 (m, 2H), 4.46–4.54 (m, 6H), 4.23–4.26 (m, 2H), 3.92 (m, 8H), 3.77–3.83 (m, 4H), 1.27 (q, 3H). ^13^C NMR ((CD_3_)_2_CO, 600 MHz): δ_C_ 194.4, 169.2, 168.3, 167.2, 138.6, 128.4, 127.6, 127.2, 66.3, 65.11, 65.10, 61.3, 58.0, 57.82, 57.80, 57.6, 57.5, 57.3, 42.5, 13.4. Elem anal. Calcd for C_23_H_30_N_4_O_8_Re^+^(TFA)(H_2_O): C, 37.95; H, 4.29; N, 6.81. Found: C, 37.99; H, 4.29; N, 6.51.

### 2.15 Synthesis of ^186^Re/^99m^Tc-Labeled Complexes

[^186^Re][ReO_4_]^−^ and [^99m^Tc][TcO_4_]^−^ were used to prepare the [^186^Re][Re(CO)_3_(OH_2_)_3_]^+^ and [^99m^Tc][Tc(CO)_3_(OH_2_)_3_]^+^ precursors following established literature procedures.^2,3^ Details for the synthesis of the precursors can be found in the Supporting Information. Both precursors were adjusted to pH 5 with 6 M HCl for radiolabeling of chelators **4**, **7**, and **8**. For radiolabeling of chelator **3**, the pH of the [^99m^Tc][Tc(CO)_3_(OH_2_)_3_]^+^ precursor was adjusted to pH 7 with 6 M HCl, and the pH of the [^186^Re][Re(CO)_3_(OH_2_)_3_]^+^ precursor was not adjusted.

All radiolabeling studies were conducted under conditions that would achieve a maximum apparent molar activity of 60–70 kBq/nmol (1.5–2 μCi/nmol) for the unpurified radiocomplexes. The conditions for radiolabeling of chelators **3**, **4**, **7**, and **8** with [^186^Re][Re(CO)_3_(OH_2_)_3_]^+^ were otherwise optimized individually. Chelator **3** (0.15 μmol in 2–7 μL of water) was combined with [^186^Re][Re(CO)_3_(OH_2_)_3_]^+^ (250 μL, pH 7, 37 MBq, 1 mCi), and the reaction mixture was diluted to 500 μL with PBS buffer (1 mM, pH 7). The reaction was heated at 95 °C for 30 min in a thermomixer. Chelator **4** (0.15 μmol in 15–20 μL of dimethyl sulfoxide) was combined with [^186^Re][Re(CO)_3_(OH_2_)_3_]^+^ (250 μL, pH 5, 37 MBq, 1 mCi), and the reaction mixture was diluted to 500 μL with MES buffer (0.2 M, pH 5). The reaction was heated at 95 °C for 30 min in a thermomixer. Chelators **7** and **8** were labeled under the same conditions as chelator **4**, except for using longer reaction times of 1 h. Attempts at direct labeling of chelators **5** and **6** with [^186^Re][Re(CO)_3_(OH_2_)_3_]^+^ proved unsuccessful. These complexes were synthesized by following the same esterification procedures as those used to synthesize **Re-5** and **Re-6**. The **[^99m^Tc]Tc-X** complexes were radiolabeled or synthesized in the same manner as that described for the **[^186^Re]Re-X** complexes.

The radiochemical yields (RCYs) were determined by radio-HPLC analyses (Methods 1 and 3), for the detection of radioactive species in solution, combined with radio-TLC analyses (Developers 1 and 2), for the detection of insoluble radioactive species (colloids).

### 2.16 *In Vitro* Stability Experiments

Radiocomplex stability testing was carried out in PBS buffer (350–400 μL) with either no challenger or l-cysteine (50 μL, 10 mM) or l-histidine (50 μL, 10 mM) as the challenger. All stability tests also contained ascorbic acid (50 μL, 1 mg/mL in PBS, pH 7) as a radioprotectant. HPLC-purified radiocomplexes ([^99m^Tc]Tc, 50 μL, 3.7–5.6 MBq, 100–150 μCi; [^186^Re]Re, 50 μL, 1.9–3.7 MBq, 50–100 μCi) were added to the stability testing solutions and incubated at 37 °C. Aliquots of each solution were taken at 1, 4, and 24 h, and the stability of the complex was determined by radio-HPLC (Methods 1 and 3) or radio-TLC (Developer 1). An additional stability time point of 48 h was taken for the [^186^Re]Re complexes. The formation of colloidal [^99m^Tc]TcO_2_ or [^186^Re]ReO_2_ was monitored at each time point by radio-TLC (Developers 1 and 2).

The radiocomplex stability was also tested in rat serum. HPLC-purified radiocomplexes ([^99m^Tc]Tc, 50 μL, 7.4–11.1 MBq, 200–300 μCi; [^186^Re]Re, 50 μL, 3.0–3.7 MBq, 80–100 μCi) were added to solutions of rat serum (400 μL, Innovative-grade U.S. Origin Sprague–Dawley Rat Serum, Innovative Research, Novi, MI) and ascorbic acid (50 μL, 1 mg/mL in PBS, pH 7). The rat serum mixtures were incubated at 37 °C. Aliquots (100–300 μL) were taken at 1, 4, and 24 h for all radiocomplexes and also at 48 h for the [^186^Re]Re complexes. The rat serum aliquots were added to a 4 times volume of acetonitrile to precipitate the rat serum proteins. The solutions were vortexed for 1 min and centrifuged for 10 min. The supernatant was carefully removed, and the pellet was washed with another portion of acetonitrile (100–500 μL). The solution was again vortexed and centrifuged, and the supernatant was removed. The activities in the combined supernatants (containing radiocomplex not bound to proteins) and the pellet (containing radiocomplex bound to proteins) were measured to determine the percentage of nonspecific protein binding for each radiocomplex. Finally, the solvent was removed from the supernatant under a stream of nitrogen, and the stability of the radiocomplexes was determined by radio-HPLC (Methods 1 and 3) after reconstitution in PBS (1 mM, pH 7.4). The formation of colloidal [^99m^Tc]TcO_2_ or [^186^Re]ReO_2_ was also monitored at each time point by radio-TLC (Developers 1 and 2).

### 2.17 log *D*_7.4_ Studies

The distribution coefficient for each HPLC-purified radiocomplex was determined using the “shake-flask” method.^8^ Each radiocomplex (500 μL in PBS, 0.4–3.7 MBq, 10–100 μCi) was added to a mixture of 4.5 mL of PBS (1 mM, pH 7.4) and 5 mL of 1-octanol. The resulting solution was vortexed for 10 min and centrifuged at 5000 rpm for 10 min. The 1-octanol and PBS layers were carefully separated, and 1 mL aliquots (*n* = 4) of each layer were counted on a HPGe detector or a NaI(Tl) well detector. The distribution coefficient was calculated by dividing the average counts in the 1-octanol layer by the average counts in the PBS layer. This experiment was repeated three times for each radiocomplex, and the results were expressed as an average of the log *D*_7.4_ values for the three experiments.

## 3 Results and Discussion

### 3.1 Synthesis of TACN-Based Chelators

The synthesis of the TACN-based chelators is shown in Scheme 1. Chelator **3** was synthesized by following an adapted literature procedure^11^ starting from TACN. To allow for asymmetrical functionalization of the TACN nitrogen atoms,^9^ TACN was first converted to TACN-orthoamide, followed by functionalization with the benzylamide arm. The overall yield for the three-step synthesis was 50%. Chelator **3** was soluble in water, methanol, and acetonitrile and was stored at RT under dry, dark conditions for up to 1 week. Chelator **4** was synthesized by reacting **3** with chloroacetic acid in water with excess NaOH at 60 °C for 4 h, resulting in an isolated yield of 60%. Chelator **4** was soluble in water, methanol, and acetonitrile and stable for several months at RT in an aqueous solution. Chelators **5** and **6** were synthesized following an adapted literature procedure^15^ by reacting chelator **3** with methyl or ethyl bromoacetate, respectively, in acetonitrile with potassium carbonate. Chelator **5** was synthesized in 46% isolated yield, was soluble in methanol and acetonitrile, and remained stable for up to 1 week when stored in a methanol solution, after which hydrolysis of the esters was observed. Chelator **6** was synthesized in 54% isolated yield, was soluble in ethanol and acetonitrile, and remained stable for up to 1 week when stored in an ethanol solution.

Chelators **7** and **8** were synthesized by hydrolyzing one of the pendant ester arms of chelators **5** and **6**, respectively. To avoid hydrolysis of both esters, the reactions were carried out in 50% methanol (**7**) or 50% ethanol (**8**) in water with dilute NaOH. When a majority (>50%) of the title compounds had been hydrolyzed, the monoacid monoester products were purified by HPLC in isolated yields up to 71% and 45% for chelators **7** and **8**, respectively. Chelator **7** was soluble in methanol, ethanol, and water, while chelator **8** was soluble in ethanol and acetonitrile. Chelators **7** and **8** were stable for 2–3 weeks at RT in an aqueous solution.

All six synthesized chelators (**3**–**8**) were isolated as yellow or colorless oils, purified by HPLC, and characterized by HRMS and ^1^H and ^13^C NMR to confirm the expected structures.

### 3.2 Synthesis of Nonradioactive Re(CO)_3_-Labeled Complexes

Due to the small masses of complexes at the radiotracer level, traditional chemical characterization is not feasible. Thus, to fully characterize the metal complexes, the chelators were first labeled with the nonradioactive Re(CO)_3_ core (Scheme 2). Because there are no nonradioactive isotopes of technetium, the rhenium complexes served as standards for both the [^186^Re]Re- and [^99m^Tc]Tc-tricarbonyl complexes. Rhenium is the third-row congener of technetium, resulting in these metals having similar chemical and physical properties. As such, rhenium and technetium were anticipated to behave similarly when bound to the TACN-based chelators. It is important to note, however, that technetium and rhenium do not have identical chemistry. For example, technetium has faster reaction kinetics and a lower reduction potential than rhenium.^6^ As a result, differences in their products and stability are sometimes observed.^16−18^

The nonfunctionalized **Re-3** complex was synthesized by reacting chelator **3** with the [Re(CO)_3_(OH_2_)_3_](NO_3_) precursor in PBS buffer (pH 7) at 75 °C for 3 h and isolated by HPLC in 35% yield. The low yield was attributed to incomplete labeling of the chelator. Only ∼50% of the ligand in solution was labeled due to the formation of a rhenium byproduct that consumed the [Re(CO)_3_(OH_2_)_3_](NO_3_) precursor. Continual heating of the reaction for up to 24 h did not increase the product yield. **Re-3** was soluble in water and remained stable for 1 month when stored under dry, dark conditions at RT.

The diacid **Re-4** complex was synthesized by reacting chelator **4** with the [Re(CO)_3_(OH_2_)_3_](NO_3_) precursor in MES buffer (pH 5) at 95 °C for 5 h. The reported p*K*_a_ values for NOTA, which bears three carboxylic acid pendant arms, are 1.96, 3.22, and 5.74.^19^ The replacement of an acid pendant arm in NOTA with an amide pendant arm (benzylamide here) is expected to decrease the remaining carboxylic acid p*K*_a_ values in **4** (also for **7** and **8**), similar to that reported for the larger but analogous DOTA chelator [2,2′,2″,2‴-(1,4,7,10-tetraazacyclododecane-1,4,7,10-tetrayl)tetraacetic acid].^20^ This supports the reasonable expectation that the selected reaction pH of 5 (only slightly acidic) was sufficiently high to ensure the presence of carboxylate anions in the reaction solution. The labeling yield was quantitative by analytical HPLC (Method 1, *t*_R_ = 14.1 min, 254 nm) with an HPLC isolated yield of 57%. **Re-4** was soluble in water and methanol and remained stable when stored under dry conditions for several months.

The diester complexes **Re-5**/**Re-6** proved more difficult to synthesize. Direct labeling of **5** and **6** with [Re(CO)_3_(OH_2_)_3_](NO_3_) using various buffers ranging from pH 3 to 9 (MES, 4-(2-hydroxyethyl)-1-piperazineethanesulfonic acid, ammonium acetate, sodium acetate, saline, or PBS) and various organic solvents (methanol, ethanol, acetonitrile, dimethyl sulfoxide, dimethylformamide, chloroform, or tetrahydrofuran), along with different temperatures (50–100 °C) and heating conditions (microwave or conventional heating in a thermomixer), was attempted to no avail. Initially, the **Re-5**/**Re-6** complexes were alternatively synthesized from **Re-3** via reaction with excess methyl (**Re-5**) or ethyl (**Re-6**) bromoacetate and excess cesium carbonate in acetonitrile at RT for 24 h, resulting in nearly quantitative conversion to **Re-5** and ∼50% conversion to **Re-6**. Due to the long reaction times and excess reagents required for the reactions to proceed, these reactions were only attempted on a small mass scale (<0.5 mg). However, the success of these reactions demonstrated that functionalization of the TACN nitrogen atoms is achievable following metal coordination.

To optimize the synthesis of **Re-5**/**Re-6**, an additional method was developed. This method involved esterification of the pendant acids on **Re-4** via reaction with thionyl chloride in dry methanol (**Re-5**) or ethanol (**Re-6**) with gentle heating (Scheme 2), yielding quantitative conversion of **Re-4** to **Re-5**/**Re-6** in 1 h (by HPLC). Due to the high yields and shorter reaction times, the esterification route was chosen as the most effective synthetic method for **Re-5**/**Re-6**. The diester complexes were soluble in methanol, ethanol, and dimethyl sulfoxide and remained stable under dry conditions at RT for 3 months, with minimal evidence of hydrolysis observed for **Re-5** (<5%) and no hydrolysis observed for **Re-6**.

The monoacid monoester complexes **Re-7**/**Re-8** were synthesized by direct labeling of chelators **7** and **8** with the [Re(CO)_3_(OH_2_)_3_](NO_3_) precursor in MES buffer (pH 5) at 95 °C for 5 h. As with the diacid chelator, a slightly acidic reaction pH was used to ensure carboxylic acid ionization in the reaction solution. The labeling yield was quantitative by HPLC for **Re-7** (Method 1, *t*_R_ = 14.8 min) and 74% for **Re-8** (Method 1, *t*_R_ = 15.8 min). The lower yield observed for **Re-8** was due to incomplete labeling of the ligand. Prolonging the reaction time may have led to a higher yield but was not attempted. The monoacid monoester complexes were soluble in methanol, ethanol, and water and remained stable for several months under dry conditions.

All nonradioactive rhenium complexes were characterized by ^1^H and ^13^C NMR, HRMS, IR, and elemental analysis. Attempts to grow X-ray-crystallographic-quality crystals were unsuccessful. The anticipated structures were confirmed by ^1^H and ^13^C NMR spectra. HRMS confirmed the anticipated masses and isotope distributions expected of the rhenium-containing complexes. The incorporation of the Re-tricarbonyl core was confirmed by the presence of the two signature strong CO stretching bands, which appeared at ∼2030 cm^–1^ (symmetrical stretching mode) and ∼1900 cm^–1^ (two overlapping antisymmetrical stretching modes) and matched previously reported Re-tricarbonyl complexes.^4,8,21^ The carbonyl shifts at 194 ppm in the ^13^C NMR spectra provided further support for Re-tricarbonyl core incorporation. Elemental analyses supported the anticipated elemental compositions, revealing the presence of TFA solvent molecules trapped within the rhenium complexes (also observed in the ^13^C NMR spectra).

The ability of the monoacid monoester chelators **7** and **8** to be efficiently labeled using the same labeling conditions as the diacid chelator **4**, coupled with the inability to directly label the diester chelators **5** and **6**, supports the hypothesis that electrostatic attraction between the negatively charged (ionized) acid group(s) and the positively charged metal tricarbonyl core aids in metal coordination by drawing the metal center toward the TACN chelator. The exchange of both pendant acid groups on the TACN backbone with esters would be expected to significantly diminish such an electrostatic attraction between the metal and chelator. Consistent with this, direct labeling of the diester chelators (**5** and **6**) was extremely difficult. When one of the pendant acid groups in the monoacid monoester chelators (**7** and **8**) was restored, the direct labeling ability was also restored. Further, labeling of the diacid chelator (**4**) proceeded more quickly than that of the monoacid monoester chelators (**7** and **8**).

Steric hindrance appears to be another factor that influenced the labeling efficiencies. Chelator **3**, with no pendant arms and therefore no possibility of carboxylate anion electrostatic attraction, was successfully labeled in moderate yield. From a steric effects perspective, the TACN nitrogen atoms on chelator **3** are the most accessible of the series to the metal center because all other chelators bear pendant arms. A steric hindrance factor is also suggested by the lower labeling yield achieved for chelator **8** (monoacid monoethyl ester), with one extra methylene group, versus chelator **7** (monoacid monomethyl ester).

It is important to note that previously reported crystal structures have shown that all three pendant carboxylic acid groups on the TACN backbone of NOTA do not participate in binding to the Re/Tc-tricarbonyl metal centers.^11^ Instead, the metal coordination sphere is filled with the three TACN backbone nitrogen atoms and the three carbon monoxide (CO) ligands. With the same donor groups available, all **Re-X** complexes reported herein were expected to coordinate in the same fashion. A comparison of the ^1^H NMR spectra obtained for **Re-X** here to those of the previously reported Re-tricarbonyl-NOTA complex^11^ revealed chemical shifts that were in good agreement, namely, those for the hydrogen atoms of the TACN ring and of the pendant arm methylene groups. Further, the methylene hydrogen atoms of the pendant arms in **Re-4** (the diacid; also for the diesters **Re-5** and **Re-6**) are observed as a singlet in the ^1^H NMR. This supports their chemical equivalence and, in turn, indicates that either both or neither of the pendant arms is coordinated to Re. Also, upon coordination of the Re(CO)_3_ core by the chelator, significant downfield shifts are observed for the hydrogen atoms of the TACN rings (e.g., the multiplets shift from 3.2–2.8 to 3.7–3.5 ppm for **4** to **Re-4** and from 3.2–2.9 to 4.0–3.8 ppm for **6** to **Re-6**). Had the pendant acid (or ester) arms been bound to Re, the TACN ring hydrogen atoms adjacent to the displaced nitrogen atoms would be expected to remain more upfield in the spectra. In short, the equivalent methylene hydrogen atoms of the pendant arms coupled with the downfield shift of all TACN ring hydrogen atoms support a metal coordination mode in which the pendant arms are not participating. They do, however, clearly play an important role in the efficient labeling of these TACN-based chelators, as discussed above.

### 3.3 Radiolabeling Studies

**Figure 1:**
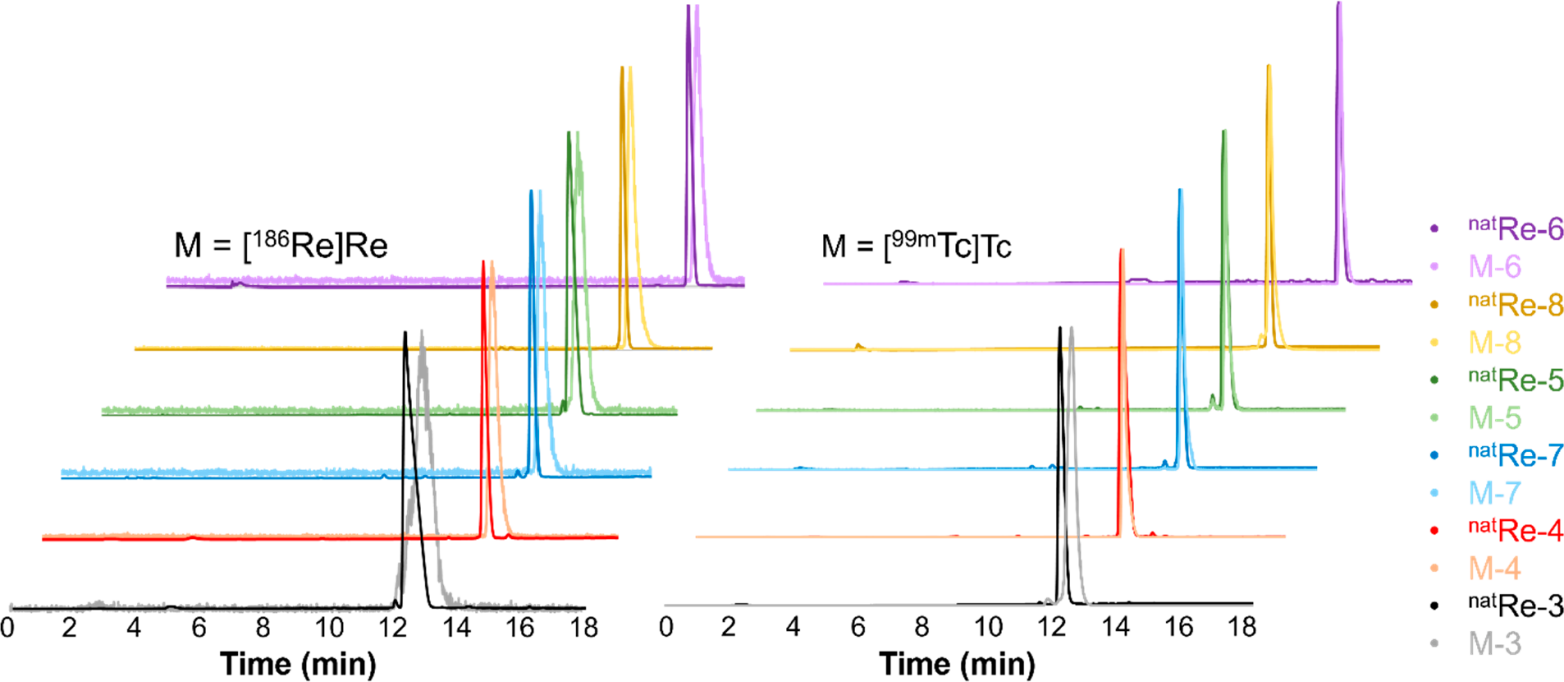
HPLC coinjections (Method 1) of the radiocomplexes with the fully characterized nonradioactive rhenium complexes.

For radiolabeling studies, the high specific activity ^99m^Tc was eluted from a ^99^Mo/^99m^Tc generator as [^99m^Tc]TcO_4_^–^ in saline. Low-specific-activity ^186^Re was produced in a nuclear reactor via the ^185^Re(n,γ)^186^Re neutron capture reaction on enriched ^185^Re targets, with ^186^Re obtained as [^186^Re]ReO_4_^–^ in saline. Radiolabeling conditions were optimized in terms of the buffer, pH, reaction time, and reaction temperature. Radiolabeling reactions were monitored by radio-HPLC and radio-TLC analyses, with no colloid formation observed for any reaction. All radioactive complexes were characterized by HPLC coinjection with their fully characterized nonradioactive rhenium complex counterparts (Figure 1), and the RCYs are given in Table 1.

**Table 1:** RCYs, Percent Stability (in PBS, l-Cysteine, and l-Histidine), and Percent Serum Protein Binding of the Radiometal Complexes^a^

		[^186^Re]Re stability^b^ (%)			
complex	[^186^Re]Re RCY (%)	PBS	l-cysteine	l-histidine	serum protein binding (%)
M-3	52 ± 3	100 ± 0	100 ± 0	100 ± 0	3 ± 2
M-4	96 ± 1	100 ± 0	100 ± 0	100 ± 0	5 ± 4
M-5	96 ± 3^c^	76 ± 2	81 ± 5	86 ± 9	3 ± 2
M-6	98 ± 3^c^	80 ± 10	85 ± 3	90 ± 10	4.8 ± 0.4
M-7	34 ± 5^d^	95 ± 2	96 ± 2	96 ± 3	6 ± 2
M-8	11.5 ± 0.5^d^	95 ± 2	94 ± 2	96 ± 1	6 ± 2

		[^99m^Tc]Tc stability^e^ (%)			
complex	[^99m^Tc]Tc RCY (%)	PBS	l-cysteine	l-histidine	serum protein binding (%)
M-3	60 ± 10	100	100	100	7 ± 0^d^
M-4	96 ± 1	100 ± 0	100 ± 0	100 ± 0	8 ± 2
M-5	93 ± 8^c^	80 ± 4^d^	84 ± 6^d^	85 ± 7^d^	6 ± 5^d^
M-6	90 ± 10^c^	80 ± 20^d^	89 ± 6^d^	91 ± 2^d^	1.7 ± 0.4^d^
M-7	19 ± 5^d^	100	100	100	4 ± 0^d^
M-8	16 ± 3^d^	100	100	100	3.5 ± 0.2^d^

a Mean values ± SD, *n* = 3.

b 48 h time points.

c RCY calculated from indirect synthesis by the esterification of **M-4**. RCY of direct-labeling reaction = 0%.

d *n* = 2.

e 24 h time points.

The nonfunctionalized chelator **3** was reacted with the [^186^Re][Re(CO)_3_(OH_2_)_3_]^+^ or [^99m^Tc][Tc(CO)_3_(OH_2_)_3_]^+^ precursor in PBS buffer (pH 7–8) at 95 °C for 30 min, resulting in RCYs of 60 ± 12% and 52 ± 3% (*n* = 3), respectively. Neutral-to-basic pH (7–9) and high temperatures were required for efficient radiolabeling. The best RCY for **[^186^Re]Re-3** was achieved at pH 7, while the best RCY for **[^99m^Tc]Tc-3** was achieved between pH values of 8 and 9. Increasing the reaction time from 30 min to 1 h did not have a significant impact on the RCY.

The diacid chelator **4** was reacted with the [^186^Re][Re(CO)_3_(OH_2_)_3_]^+^ or [^99m^Tc][Tc(CO)_3_(OH_2_)_3_]^+^ precursor in MES buffer (pH 5) at 95 °C for 30 min, resulting in RCYs of 96 ± 1% and 98 ± 1% (*n* = 3), respectively. Radiolabeling reactions in MES buffer ranging from pH 4 to 6 all resulted in high yields, with pH 5 giving the best yield. These findings are consistent with the reactions being accelerated by electrostatic attraction because the highest RCYs were achieved at a reaction pH that was higher than 2 of the reported NOTA p*K*_a_ values and near the third.^19^ The ^186^Re radiolabeling of **4** was tested under varying temperature conditions ranging from 50 to 95 °C, with the expected lower RCYs observed at lower reaction temperatures. For example, at 50 °C, the reaction was allowed to proceed for 24 h, resulting in an RCY of 76%. At 65 and 75 °C, reaction completion (i.e., consumption of the [^186^Re][Re(CO)_3_(OH_2_)_3_]^+^ precursor via either radiolabeling of the chelator or oxidation of the precursor) was achieved in 3 h, with RCYs of 87% and 98%, respectively. Quantitative RCYs were achieved in 1 h at 85 °C and in 30 min at 95 °C. Due to the shorter half-life of ^99m^Tc, radiolabeling of the **[^99m^Tc]Tc-4** complex was only analyzed at 1 h under different temperature conditions. The RCYs were 13%, 52%, and 72% at 50, 65, and 75 °C, respectively. The highest RCYs of 83 ± 4% and 98 ± 1% were achieved at 85 and 95 °C, respectively, with a quantitative RCY of **[^99m^Tc]Tc-4** achieved in as little as 15 min at 95 °C. Relatively high ligand concentrations of 0.2–0.4 mM were needed to achieve quantitative radiolabeling yields.

Given the known faster reaction kinetics for technetium versus rhenium, direct labeling of the dimethyl ester chelator **5** was attempted with the [^99m^Tc][Tc(CO_3_)(OH_2_)_3_]^+^ precursor. As with the rhenium labeling studies, no labeling was observed despite a wide range of pH, buffer, temperature, and time conditions used. Assuming similar results would be obtained for the diethyl ester chelator **6**, direct labeling of chelator **6** was not attempted.

Following the synthesis used for **Re-5**, the syntheses of dimethyl esters **[^186^Re]Re-5** and **[^99m^Tc]Tc-5** were first attempted by reacting HPLC-isolated **[^186^Re]Re-3** and **[^99m^Tc]Tc-3** with an excess of cesium carbonate and methyl bromoacetate at RT for 18 h. HPLC analysis revealed that no **[^186^Re]Re-5** or **[^99m^Tc]Tc-5** had formed, suggesting that the reactions are not feasible at the low concentrations used for the radioactive complex syntheses. Instead, the diester complexes **[^186^Re]Re-5/6** and **[^99m^Tc]Tc-5/6** were synthesized via the esterification method by reaction of **[^186^Re]Re-4** and **[^99m^Tc]Tc-4** with dry methanol or ethanol, respectively, and thionyl chloride for 1 h at 60 °C. HPLC analyses revealed >90% RCYs with ≤10% radiometal oxidation to the permetallate (7+ oxidation state) during the reaction.

The monoacid monomethyl ester chelator **7** was reacted with the [^186^Re][Re(CO)_3_(OH_2_)_3_]^+^ precursor in MES buffer. The reaction was successfully carried out in a pH range of 3–6, with pH 5 resulting in the highest **[^186^Re]Re-7** average yield of 34 ± 5%. The low reaction yield was due to simultaneous formation of the **[^186^Re]Re-4** product, from hydrolysis of the single ester arm on chelator **7** to an acid (giving chelator **4**) with subsequent radiolabeling and/or from hydrolysis of the labeled **[^186^Re]Re-7** product during the reaction. Under the optimized radiolabeling conditions, the ratio of **[^186^Re]Re-7** to **[^186^Re]Re-4** in the final product was 1:1. The radiolabeling was carried out at temperatures ranging from 50 to 95 °C. The reactions at lower temperatures (50–75 °C) all resulted in RCYs of <10% through 1 h, with a minimal increase in yield with prolonged heating. Increasing the temperature to 85 °C resulted in a RCY of 17 ± 5% in 1 h (*n* = 3), and the highest RCY of 34 ± 5% (*n* = 2) was achieved at 95 °C in 1 h. Increasing the reaction time from 1 to 2 h had little to no effect on the RCY.

Similar results were observed for the **[^99m^Tc]Tc-7** complex. The highest RCY, 19 ± 5% (*n* = 2), was achieved by reacting chelator **7** with the [^99m^Tc][Tc(CO)_3_(OH_2_)_3_]^+^ precursor in MES buffer (pH 5) at 95 °C for 1 h. The chelator was successfully radiolabeled in the pH range of 3–6 with no significant difference in yield. The hydrolysis product, **[^99m^Tc]Tc-4**, was also observed, although to a lesser extent than that with the radiorhenium complex. The ratio of **[^99m^Tc]Tc-7** to **[^99m^Tc]Tc-4** after a 1 h reaction time at 95 °C was 2:1. Reducing the ligand concentration from 0.3 to 0.1 mM decreased the amount of hydrolysis product generated, nearly stopping its formation; however, a concomitant loss in **[^99m^Tc]Tc-7** RCY was also observed (7% RCY). Increasing the ligand concentration to 1 mM increased the yield of **[^99m^Tc]Tc-7** to 50% with a ratio of **[^99m^Tc]Tc-7** to **[^99m^Tc]Tc-4** of 2:1.

Chelator **8** was reacted with the [^186^Re][Re(CO)_3_(OH_2_)_3_]^+^ precursor in MES buffer (pH 5) at 95 °C for 1 h, resulting in an RCY of 11.5 ± 0.5% (*n* = 2). Increasing the reaction time to 2 h increased the RCY to 17%. Hydrolysis of the chelator to **4** was also observed. Under the optimized reaction conditions, the ratio of **[^186^Re]Re-8** to **[^186^Re]Re-4** was 1.25:1. The RCY for the **[^99m^Tc]Tc-8** complex was 16 ± 3% (*n* = 2) under the optimized conditions, with a similar ratio of **[^99m^Tc]Tc-8** to **[^99m^Tc]Tc-4**. Increasing the ligand concentration to 1 mM resulted in a RCY of 28%.

A trade-off to the kinetic inertness of Tc^I^/Re^I^-tricarbonyl radiocomplexes (*vide infra*) is the slower kinetics of ligand exchange that leads to their formation.^7^ As demonstrated above, this can be compensated for by using higher ligand masses and higher reaction temperatures. Using higher ligand masses has its own trade-off, namely, lowered radiocomplex apparent molar activities, which can, in turn, be addressed by the HPLC separation of the radiolabeled product from the excess ligand. This is easily accomplished due to the lipophilic nature of the metal tricarbonyl core and the associated significant difference in HPLC retention times between the unlabeled ligand and radiocomplex product. Using elevated reaction temperatures has the downside of precluding the use of the M^I^(CO)_3_^+^ radiolabeling approach in certain applications, such as with biomolecule conjugates that are heat-sensitive.

### 3.4 *In Vitro* Stability

The stability of the radiocomplexes was tested in PBS buffer, l-cysteine, l-histidine, and rat serum at 37 °C. The stability of each complex was evaluated by radio-HPLC and/or radio-TLC through 24 h for all [^99m^Tc]Tc complexes and through 48 h for all [^186^Re]Re complexes (Table 1).

The nonfunctionalized **M-3** and diacid **M-4** (M = [^99m^Tc]Tc or [^186^Re]Re) complexes remained stable through 24 or 48 h under all tested conditions. No colloidal MO_2_ or permetallate ions were observed by radio-TLC or radio-HPLC, respectively. The **M-3** complexes demonstrated less than 5% protein binding across all time points, while the **M-4** complexes demonstrated between 5 and 10% protein binding. High stability of the **M-3** and **M-4** complexes was expected because TACN and NOTA bifunctional chelators that were conjugated to gastrin-releasing peptide receptor targeted peptides and radiolabeled with ^99m^Tc(CO)_3_ were shown to be stable in previous *in vitro*(21,22) and *in vivo*(12) experiments.

While the complexes bearing pendant ester arms showed some reduction in stability, the observed degradation was exclusively due to hydrolysis of the pendant ester arm(s) over time. Importantly, no colloidal MO_2_ or permetallate ions were observed, demonstrating the highly stable coordination of the M(CO)_3_ core by the TACN-based chelators. The overall stability of these complexes was thus limited only by the stability of their pendant functional groups. Sequential hydrolysis of the ester arms was observed for **M-5** and **M-6**, leading first to **M-7** and **M-8** intermediates and eventually to the formation of **M-4**.

All ester derivative complexes demonstrated high stability in PBS, l-cysteine, and l-histidine. Complexes **[**^**186**^**Re]Re-5** and **[**^**186**^**Re]Re-6** remained >95% stable through 4 h, with >76% of the complexes left intact after 48 h. The **[**^**99m**^**Tc]Tc-5** and **[**^**99m**^**Tc]Tc-6** complexes demonstrated >90% stability through 4 h and >79% stability through 24 h. The **[**^**186**^**Re]Re-7** and **[**^**186**^**Re]Re-8** complexes demonstrated >94% stability through 48 h, while the **[**^**99m**^**Tc]Tc-7** and **[**^**99m**^**Tc]Tc-8** complexes were 100% stable through 24 h.

**Figure 2:**
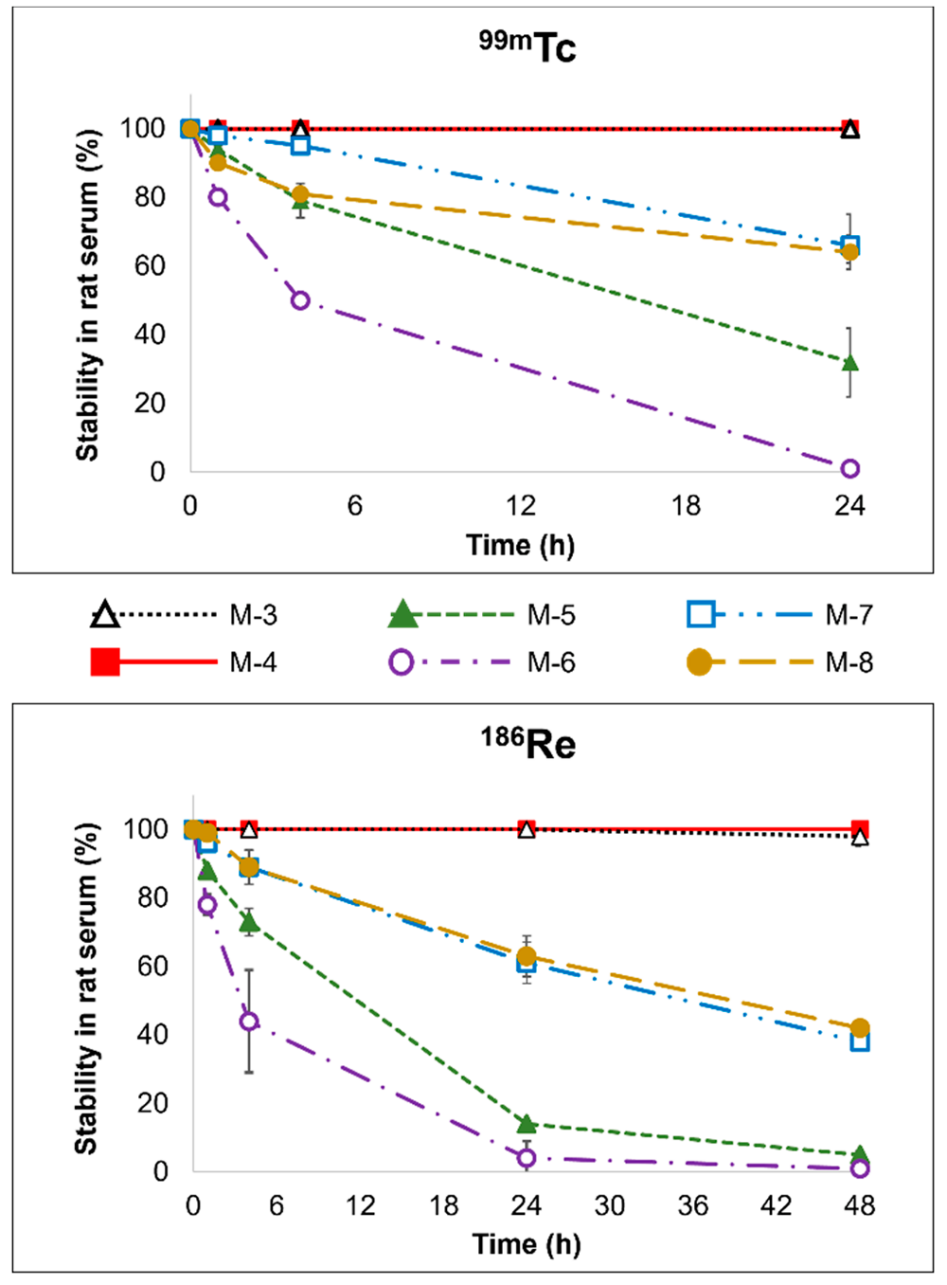
Radiocomplex stability in rat serum. HPLC-isolated radiocomplexes were incubated in rat serum at 37 °C for 24 h (^99m^Tc) or 48 h (^186^Re). At given time points, the radiocomplex was analyzed by radio-HPLC and/or radio-TLC to evaluate stability. All decomposition observed was due only to hydrolysis of the pendant ester arm(s). No permetallate (MO_4_^–^) or colloid (MO_2_) formation was observed.

For peptide-based radiopharmaceutical development, the first 4 h postinjection *in vivo* is the most important because the radiopharmaceutical is typically expected to accumulate at the targeted site or to be processed via the clearance organs during that time. The ester complexes (**M-5/6/7/8**) showed promising stability through 4 h in nonbiological solutions, while their stability in rat serum was reduced (Figure 2). At 1 h, the stabilities of the dimethyl ester **[**^**186**^**Re]Re-5** and **[**^**99m**^**Tc]Tc-5** complexes in rat serum were 88 ± 1% and 94 ± 1%, respectively, which decreased over time to a final average stability of 4 ± 1% (*n* = 3) at 48 h for **[**^**186**^**Re]Re-5** and 32 ± 10% (*n* = 2) at 24 h for **[**^**99m**^**Tc]Tc-5**. For the diethyl ester **[**^**186**^**Re]Re-6** and **[**^**99m**^**Tc]Tc-6** complexes, ≤2% remained intact through 48 and 24 h, respectively. For all **M-5/6** complexes the protein binding was <6%.

The monoacid monoester **M-7/8** complexes showed improved stability in rat serum relative to the diester derivatives. The **[**^**186**^**Re]Re-7** and **[**^**186**^**Re]Re-8** complexes demonstrated 38% and 42% stability through 48 h, respectively, while the **[**^**99m**^**Tc]Tc-7** and **[**^**99m**^**Tc]Tc-8** complexes remained >60% stable in rat serum through 24 h (Figure 2). For all **M-7/8** complexes, the protein binding was <6%.

### 3.5 log *D*_7.4_ Studies

**Figure 3:**
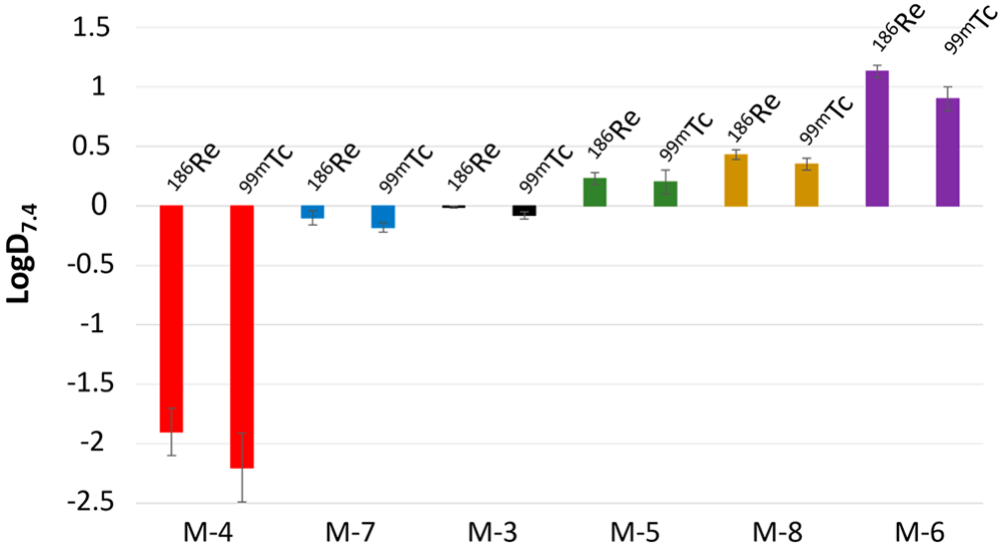
log *D*_7.4_ values of the radiocomplexes determined using the “shake-flask” method. HPLC-isolated radiocomplexes were combined and vortexed in a mixture of octanol and PBS (pH 7.4). After centrifugation, the octanol and PBS layers were separated and counted on a NaI(Tl) or HPGe detector. The log *D*_7.4_ values were calculated using the equation log *D*_7.4_ = log(counts in octanol/counts in PBS).

The log *D*_7.4_ values for the radiocomplexes were evaluated by PBS and 1-octanol partitioning to measure the hydrophilicity of each complex (Figure 3). As expected, the diacid **M-4** complexes demonstrated the greatest hydrophilic character, with log *D*_7.4_ values around −2. The next most hydrophilic complexes were the monoacid monomethyl ester **M-7** and nonfunctionalized **M-3** complexes, with log *D*_7.4_ values between −0.2 and 0. The remaining three sets of complexes (the diesters **M-5** and **M-6** and monoacid monoethyl ester **M-8**) yielded positive log *D*_7.4_ values ranging from +0.2 to +1.1, indicating lipophilic character. Only small differences in the log *D*_7.4_ values were observed between the [^99m^Tc]Tc- and [^186^Re]Re-radiolabeled complexes, with the [^99m^Tc]Tc complexes showing slightly more hydrophilic character.

For radiopharmaceutical development, hydrophilic complexes are often preferred for their faster clearance via renal–urinary excretion, leading, in turn, to higher imaging contrast and lower healthy tissue radiation exposure. Thus, considering both their lipophilic character and hydrolytic tendency, the diester chelators are not well suited for radiopharmaceutical development. Pairing the slightly hydrophilic complexes **M-7** and **M-3** with a sufficiently hydrophilic targeting vector (and/or linking molecule) may result in acceptable pharmacokinetics and clearance, although hydrolysis of the ester in **M-7***in vivo* would need to be carefully evaluated. As noted above, this set of chelators was not selected specifically for radiopharmaceutical development but instead to explore relatively small structural changes that were expected to have significant (radio)chemistry impacts.

## 4 Conclusions

Six TACN-based ligands bearing acid, ester, mixed acid–ester, or no pendant functional groups were successfully synthesized, characterized, and (radio)labeled with the [M(CO)_3_]^+^ cores (M = Re, ^186^Re, and ^99m^Tc). The resulting metal tricarbonyl complexes were remarkably stable, although those bearing pendant ester arms did undergo hydrolysis of the esters over time. The diacid chelator **4** (radio)labeled the fastest with the highest overall yields, followed by the nonfunctionalized chelator **3** and the monoacid monoester chelators **7** and **8**. Direct labeling of the diester chelators **5** and **6** with the [M(CO)_3_(OH_2_)_3_]^+^ precursor was not successful; however, their M(CO)_3_-labeled complexes were synthesized by esterification of the **M-4** complexes. Although the TACN pendant groups do not participate in coordination of the metal center, they certainly impact the M(CO)_3_ (radio)labeling reaction efficiency. In these studies, the number of ionizable pendant acid arms correlated with the (radio)labeling yields of the functionalized chelators, supporting the hypothesis that electrostatic attraction between the negatively charged pendant functional groups and the positively charged metal tricarbonyl core aids (radio)labeling. Future development of TACN-based chelators for (radio)labeling with the [M(CO)_3_]^+^ cores will probe additional modified chelators, including those bearing other ionizable functional groups.

## Supporting Information Available

Synthetic procedures, ^1^H and ^13^C NMR for chelators and rhenium complexes, and IR data for rhenium complexes (PDF)
