# Supplementary material for: 1,4,7-Triazacyclononane-Based Chelators for the Complexation of [186Re]Re- and [99mTc]Tc-Tricarbonyl Cores
Source: Inorg Chem. 2023 Sep 8;62(50):20688–98. doi: 10.1021/acs.inorgchem.3c01934 (PMC10732151; doi:10.1021/acs.inorgchem.3c01934)
Supplement: Supplementary file 1 — ic3c01934_si_001.pdf [file ic3c01934_si_001.pdf]

Supporting Information

1,4,7-Triazacylononane-Based Chelators for the Complexation of [^{186}Re]Re- and [^{99m}Tc]Tc-Tricarbonyl Cores

Rebecca Hoerres[†] and Heather M. Hennkens[*,†,‡]

[†] Department of Chemistry, University of Missouri, Columbia, Missouri 65211, United States

[‡] Research Reactor Center, University of Missouri, Columbia, Missouri 65211, United States

*Email: HennkensH@missouri.edu.

Table of Contents:

1. Synthetic procedures -- pp 2-3

2. NMR Spectra -- pp 4-18

3. IR Spectra -- pp 19-20

4. References --- p 21

1. Synthetic procedures:

1.1 General

Liquid chromatography electrospray ionization mass spectrometry (LCMS) analyses were performed on a HPLC Gold System (Beckman Coulter, Fullerton, CA). The HPLC was coupled to an ion trap mass spectrometer (LCQ Fleet from Thermo Fisher, Waltham, MA). A Thermo Fisher Scientific BetaBasic C18 column (150 mm x 4.6 mm, 5 µm) was used with a binary linear gradient of 30% to 50% acetonitrile (with 0.1% TFA) in water (with 0.1% TFA) over 30 min (Method 5) and a flow rate of 1 mL/min. LCMS chromatograms were analyzed at the 254 nm wavelength.

1.2 Synthesis of *N*-benzyl-2-bromoacetamide

N-benzyl-2-bromoacetamide was synthesized as previously reported.[1] Benzylamine (2.5 g, 0.023 mol) was added to 10 mL of dichloromethane and cooled in an ice bath. Bromoacetyl bromide (2.3 g, 0.011 mol) was added dropwise over 5 minutes, resulting in the immediate formation of a white precipitate. The reaction was allowed to warm to room temperature and stir for 3 h. The reaction mixture was filtered, and the colorless solution was dried under reduced pressure to yield *N*-benzyl-2-bromoacetamide as a white powder. Isolated yield: 76% (2.0 g). The product was characterized by HRMS, ^{1}H NMR, and ^{13}C NMR. HRMS (m/z): calculated for $C_9H_{10}BrNO$, $[M+H]^+$ 228.0019, found 228.0001. ^{1}H NMR (CD_2Cl_2, 600 MHz): δ_H 7.38-7.40 (m, 2H), 7.32-7.34 (m, 3H), 6.80 (s, 1H), 4.49 (d, 2H), 3.94 (s, 2H). ^{13}C NMR (CD_2Cl_2, 600 MHz): δ_C 165.3, 137.8, 128.7, 127.6, 43.9, 29.3.

1.3 Synthesis of TACN-orthoamide (1)

Compounds **1** and **2** were synthesized as previously reported.[1] For compound **1**, TACN (167 mg, 1.3 mmol) was dissolved in 3 mL of dry acetonitrile. *N*,*N*-Dimethylformamide dimethyl acetal (154 mg, 1.3 mmol) was added, and the reaction mixture was heated at 80 °C for 3 h in an oil bath. The solvent was removed under reduced pressure to yield **1** as a yellow oil. The product was used without further purification. Yield: 93% (165 mg). The product was characterized by ^{1}H NMR and ^{13}C NMR. ^{1}H NMR ($CDCl_3$, 500 MHz): δ_H 4.99 (s, 1H), 3.00-3.07 (m, 6H), 2.72-2.83 (m, 6H). ^{13}C NMR ($CDCl_3$, 500 MHz): δ_C 103.9, 51.6.

1.4 Synthesis of *N*-benzyl-2-(1,4,7-triazonaneorthoamidyl)acetamide (2)

Compound **1** (165 mg, 1.2 mmol) was dissolved in 5 mL of dry tetrahydrofuran. *N*-benzyl-2-bromoacetamide (300 mg, 1.3 mmol) in 5 mL of dry tetrahydrofuran was added, and the reaction was stirred at room temperature for 18 h. The reaction was filtered to recover the product as a yellow oil, which was then HPLC purified in 10-15 mg batches by semi-preparative HPLC (Method 2, t_R = 9.2 min). The HPLC eluate was dried under reduced pressure to yield **2** as a brown oil. Isolated yield: 61% (210 mg). The product was characterized by LCMS, ^{1}H NMR, and ^{13}C NMR. LCMS (Method 5, t_R = 8.3 min) (m/z): calculated for $C_{16}H_{23}N_4O^+$, $[M]^+$ 287.19, found 305.18 corresponding to the formamide product (Scheme 1, 2f; calculated $[M]^+$ 305.19). Two

isomers of the formamide product are visible in the NMR spectra due to the slow rotation around the C-N amide bond.[2] Chemical shifts for only one isomer are listed. ^{1}H NMR (CD_3CN, 500 MHz): δ_H 8.07 (s, 1H), 7.36-7.39 (m, 2H), 7.29-7.32 (m, 3H), 7.25 (s, 1H), 4.42 (d, 2H), 3.72 (t, 2H, *J* = 5.3 Hz), 3.55 (s, 2H), 3.48 (t, 2H, *J* = 5.3 Hz), 3.32 (t, 2H, *J* = 4.7 Hz), 3.14 (t, 2H, *J* = 5.7 Hz), 2.91 (t, 2H, *J* = 4.7 Hz), 2.76 (t, 2H, *J* = 5.7 Hz). ^{13}C NMR (CD_3CN, 500 MHz): δ_C 173.4, 165.2, 138.4, 128.5, 127.4, 127.3, 55.7, 53.8, 51.4, 48.7, 47.0, 46.1, 43.6, 42.9.

1.5 Synthesis of $(NEt_4)_2[Re(CO)_3Br_3]$

$(NEt_4)_2[Re(CO)_3Br_3]$ was synthesized according to a literature procedure.[3] Tetraethylammonium bromide (570 mg, 2.9 mmol) was dissolved in 20 mL of diglyme and heated to 80 °C in an oil bath. Rhenium(I) pentacarbonyl bromide (500 mg, 1.2 mmol) in 20 mL of diglyme was added. The temperature of the reaction was increased to 115 °C and heated for 4 h under a nitrogen atmosphere. During the reaction, a white precipitate formed. The precipitate was filtered and washed with fresh diglyme (10 mL) and cold diethyl ether (10 mL), then slurried in ethanol to remove excess tetraethylammonium bromide and subsequently filtered to give the product, $(NEt_4)_2[Re(CO)_3Br_3]$, as a white powder. Isolated yield: 84% (801 mg). The CO ligand stretching bands were observed by IR spectroscopy at 1847 cm^{-1} and 1996 cm^{-1}, matching the values reported in literature.[3]

1.6 Synthesis of ^{186}Re/^{99m}Tc-labeled complexes

The $[^{186}Re][Re(CO)_3(OH_2)_3]^+$ precursor was synthesized according to a literature procedure.[4] A kit containing borane-ammonia complex (5 mg) was purged with carbon monoxide for 20 minutes. A solution containing $[^{186}Re][ReO_4]^-$ (74-148 MBq, 2-4 mCi) and 85% phosphoric acid (7 µL) in saline (1 mL) was added to the kit via syringe. The reaction was heated at 65 °C for 15 minutes in a water bath, during which the pressure of the sealed vial was balanced with a 20 mL syringe. After cooling, the kit was opened and the product, $[^{186}Re][Re(CO)_3(OH_2)_3]^+$, was analyzed by radio-HPLC (Method 1, t_R = 3.1 and 4.8 min). The product presents as two peaks, as reported previously,[5] both of which react with suitable chelators.

The $[^{99m}Tc][Tc(CO)_3(OH_2)_3]^+$ precursor was synthesized according to a literature procedure.[6] A kit containing sodium borohydride (7 mg), sodium carbonate (4 mg), and potassium sodium tartrate tetrahydrate (15 mg) was purged with carbon monoxide for 20 minutes. A solution containing $[^{99m}Tc][TcO_4]^-$ (185-370 MBq, 5-10 mCi) in saline (1 mL) was added to the kit via syringe. The reaction was heated in a boiling water bath for 15 min. After heating, the kit was vented with a 20 mL syringe and opened. The product, $[^{99m}Tc][Tc(CO)_3(OH_2)_3]^+$, was analyzed by radio-HPLC (Method 1, t_R = 3.7 min).

2. NMR Spectra

^{1}H and ^{13}C NMR spectra were acquired on a Bruker Avance III 500 MHz or 600 MHz spectrometer. The NMR data were analyzed with Bruker TopSpin version 4.0.9.

2.1 ^{1}H NMR *N*-benzyl-2-bromoacetamide (600 MHz, CD_2Cl_2)

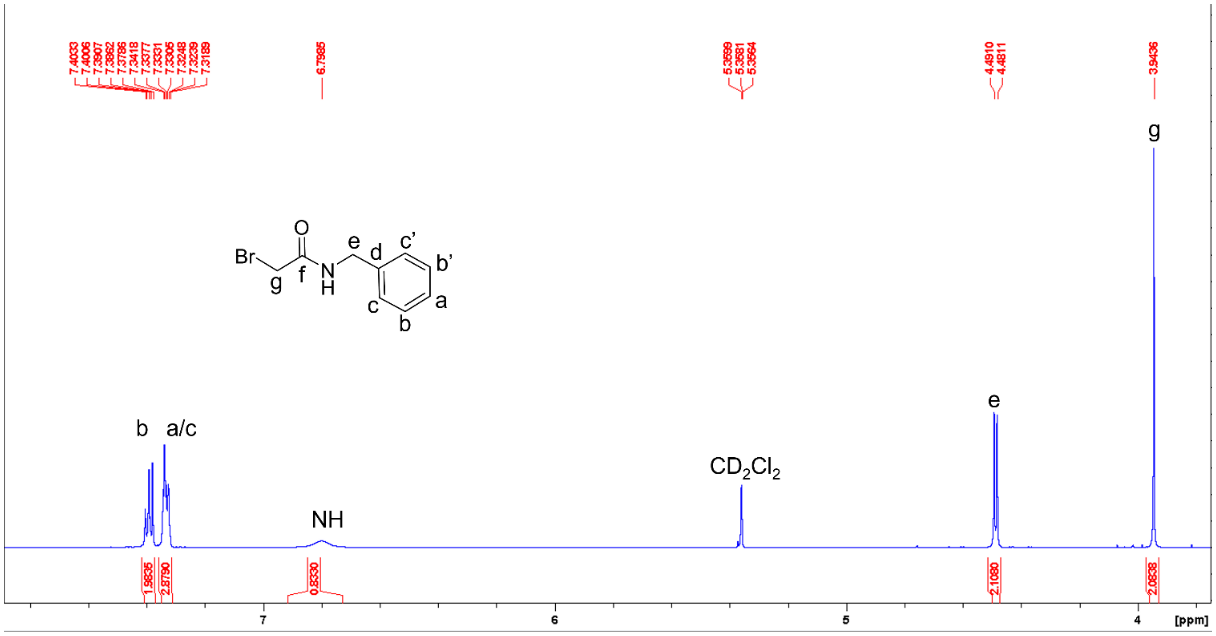

2.2 ^{13}C NMR *N*-benzyl-2-bromoacetamide (600 MHz, CD_2Cl_2)

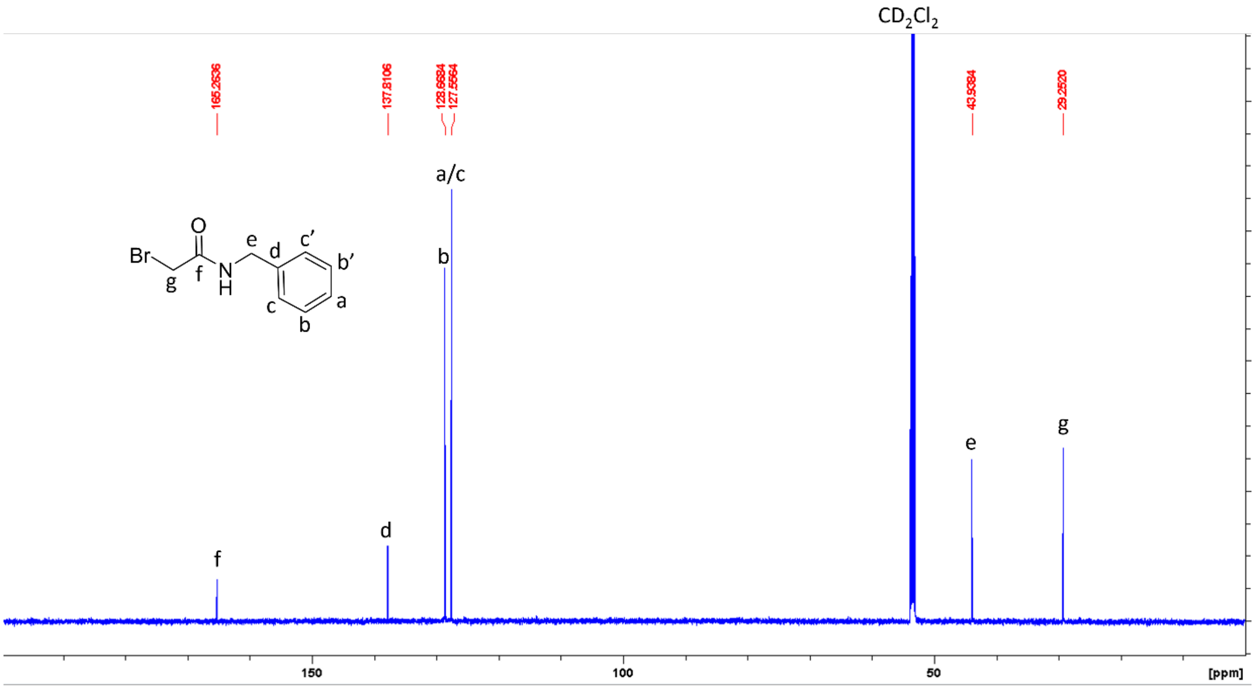

2.3 ^{1}H NMR **1** (500 MHz, $CDCl_3$)

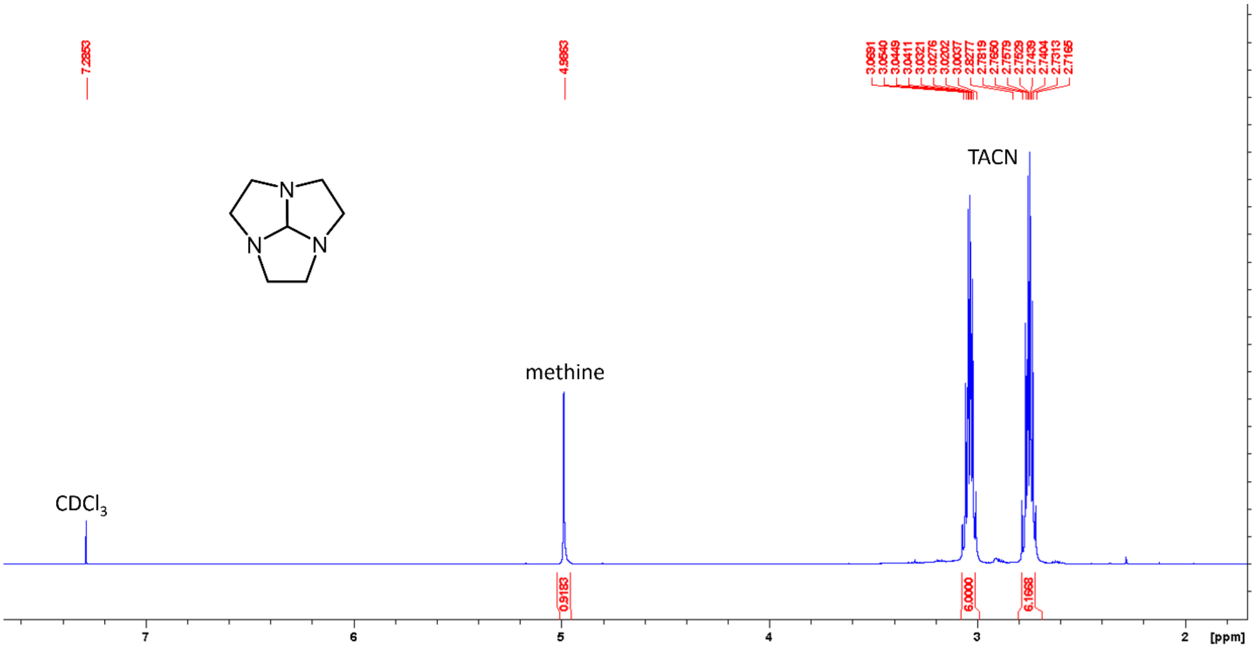

2.4 ^{13}C NMR **1** (500 MHz, $CDCl_3$)

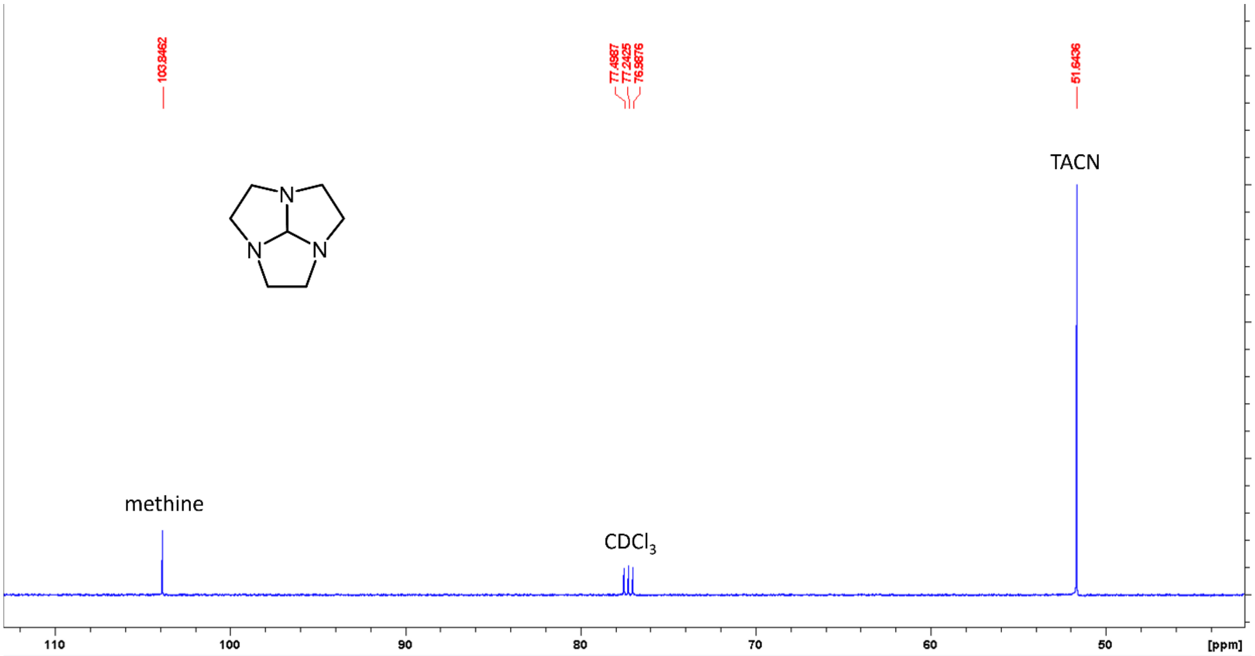

2.5 ^{1}H NMR **2** (500 MHz, CD_3CN)

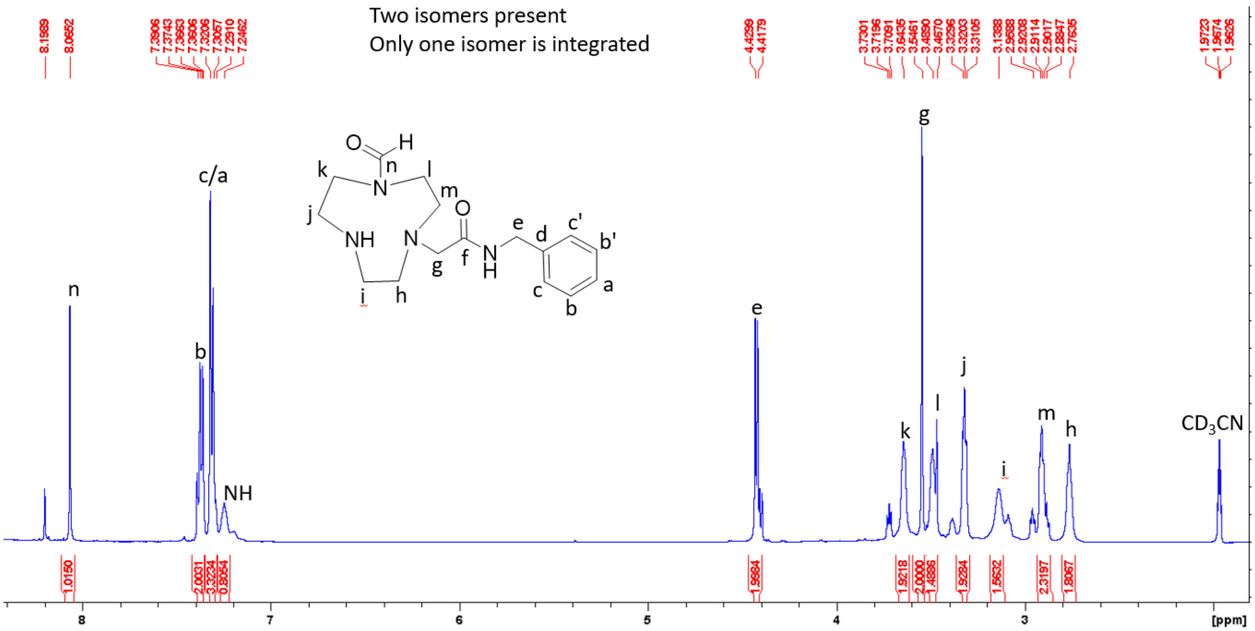

2.6 ^{13}C NMR **2** (500 MHz, CD_3CN)

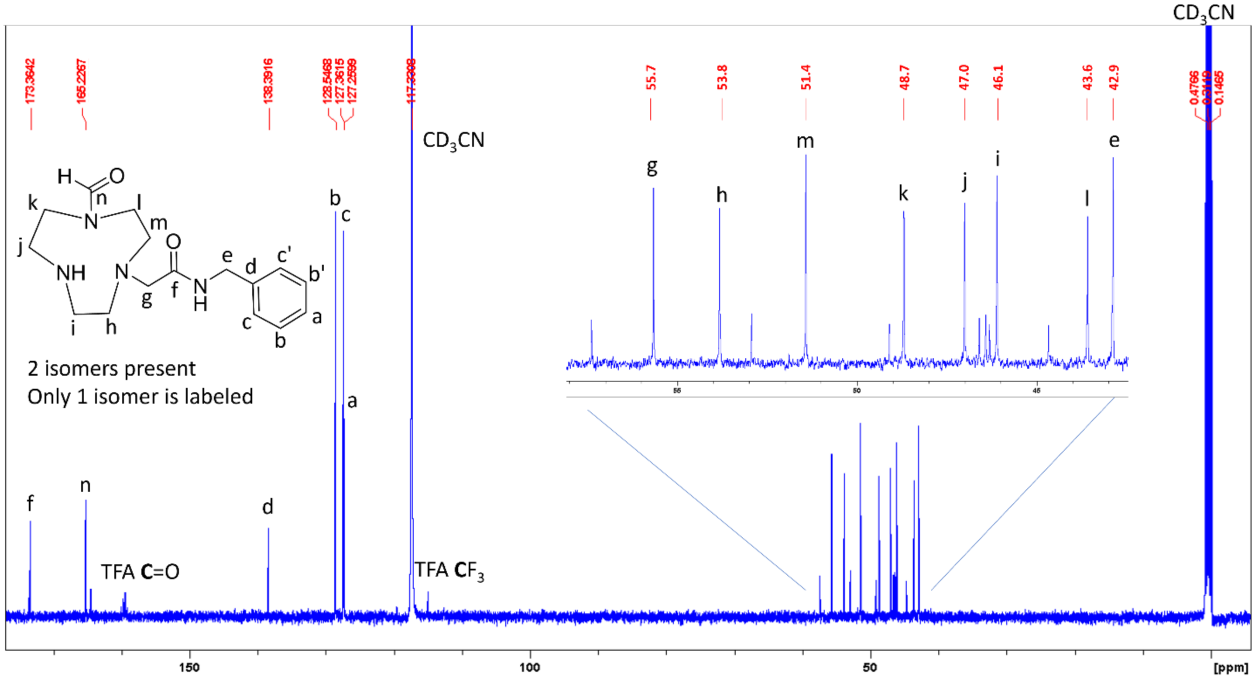

2.7 ^{1}H NMR **3** (600 MHz, D_2O)

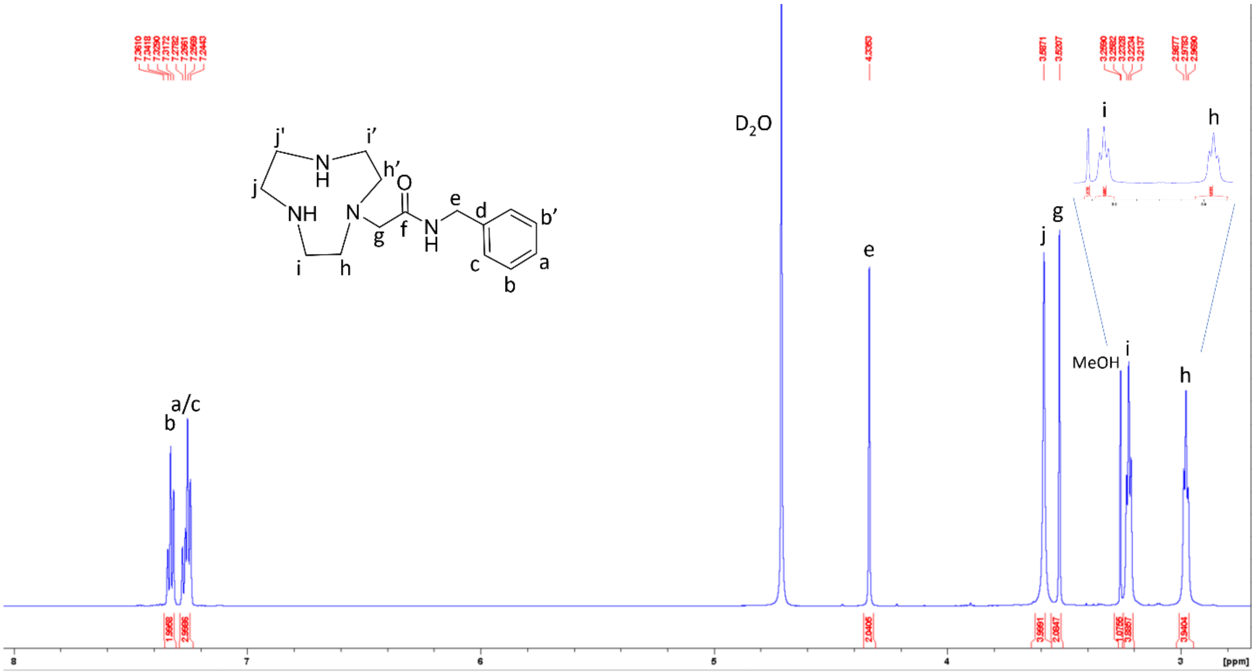

2.8 ^{13}C NMR **3** (600 MHz, D_2O)

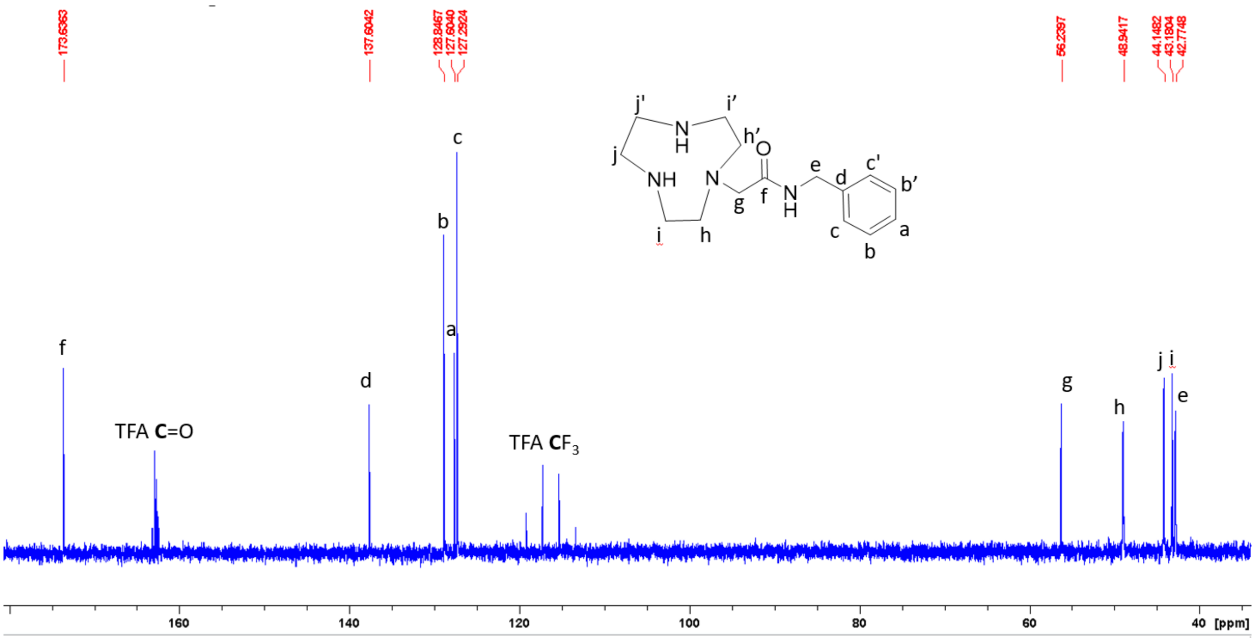

2.9 ^{1}H NMR **4** (600 MHz, $(CD_3)_2SO$)

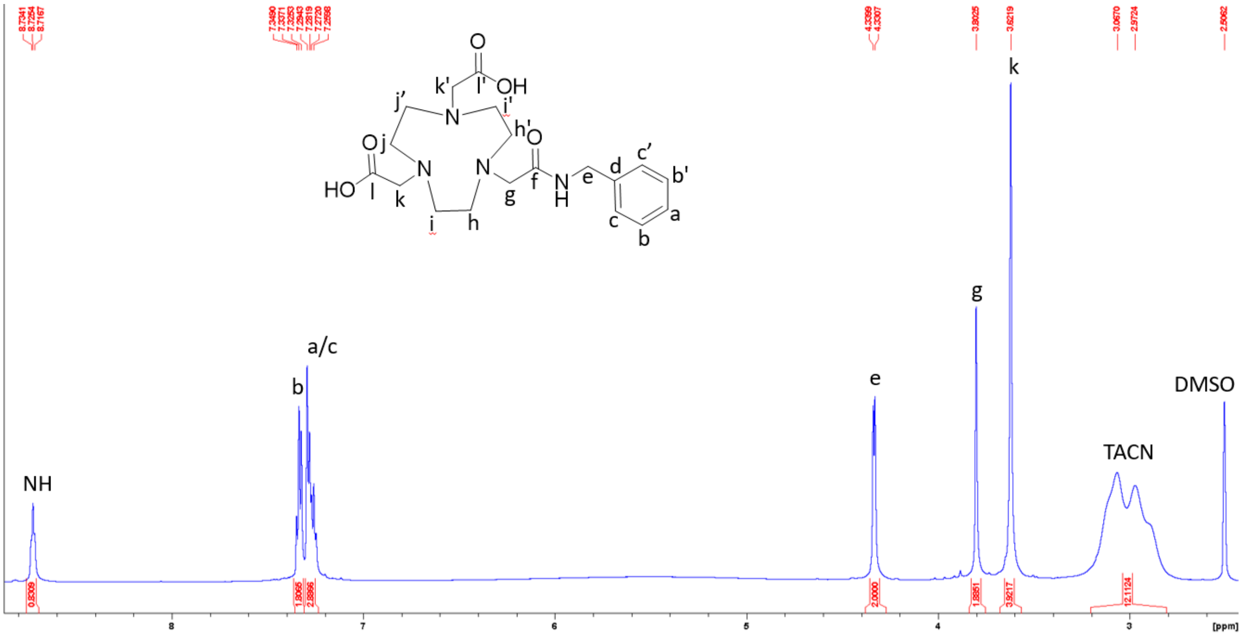

2.10 ^{13}C NMR **4** (600 MHz, (CD_3CN)

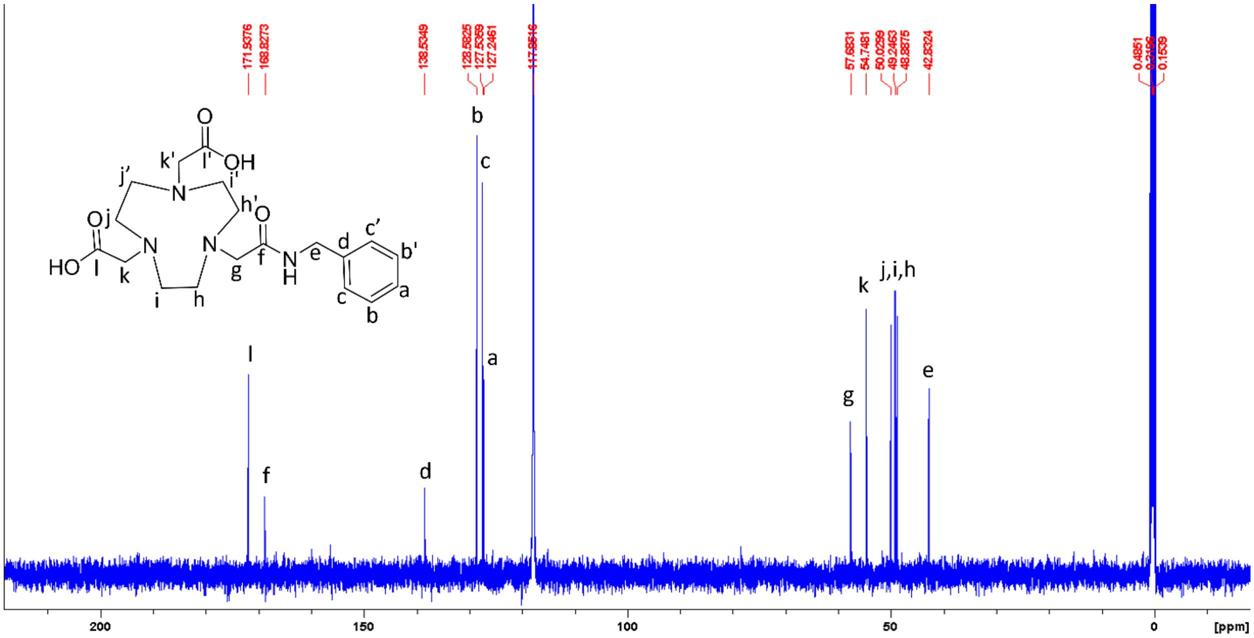

2.11 ^{1}H NMR **5** (600 MHz, $CDCl_3$)

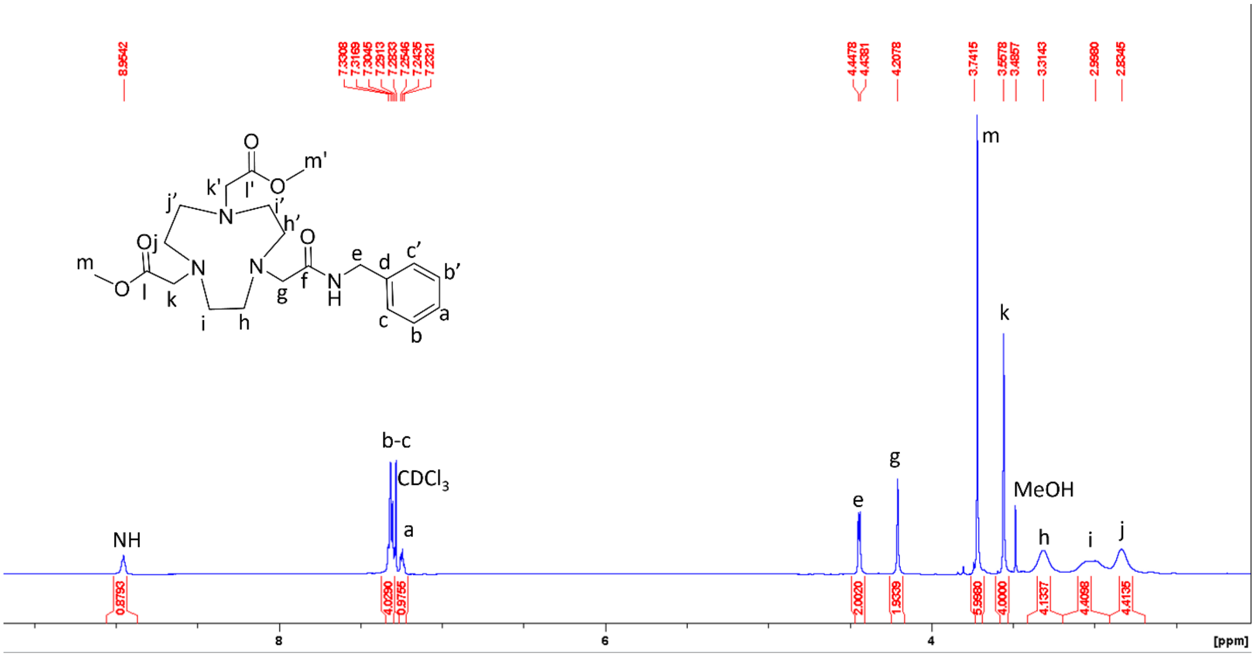

2.12 ^{13}C NMR **5** (600 MHz, $CDCl_3$)

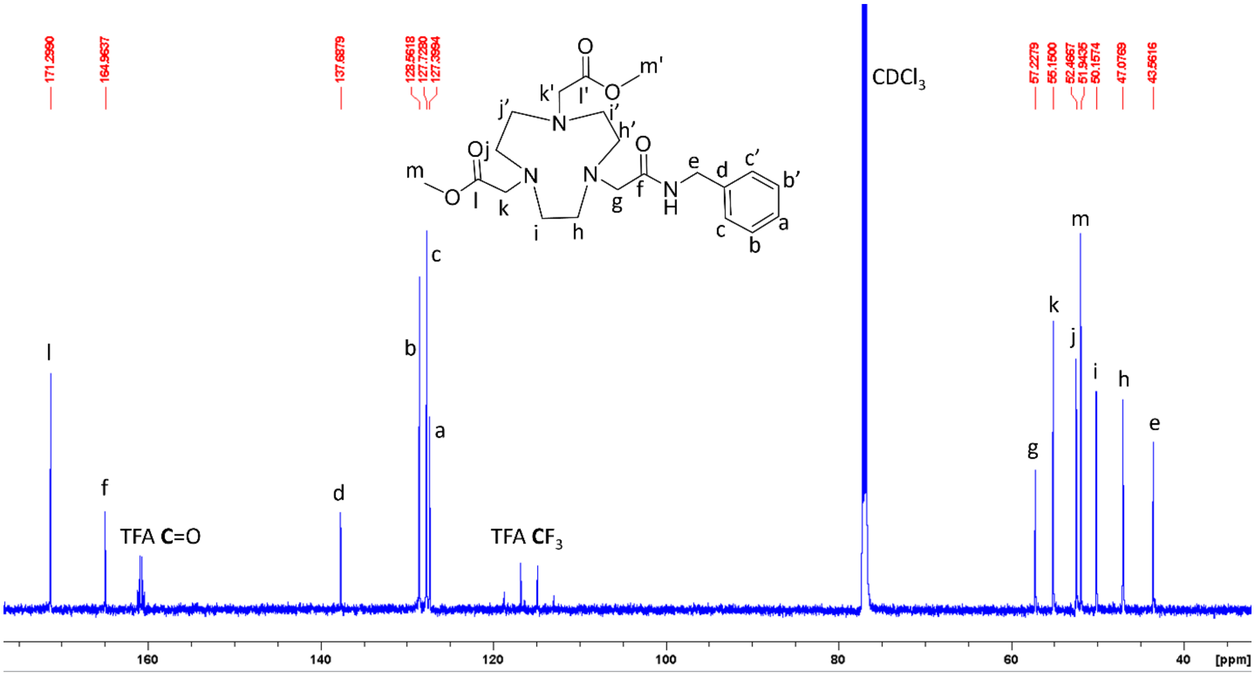

2.13 ^{1}H NMR **6** (600 MHz, $CDCl_3$)

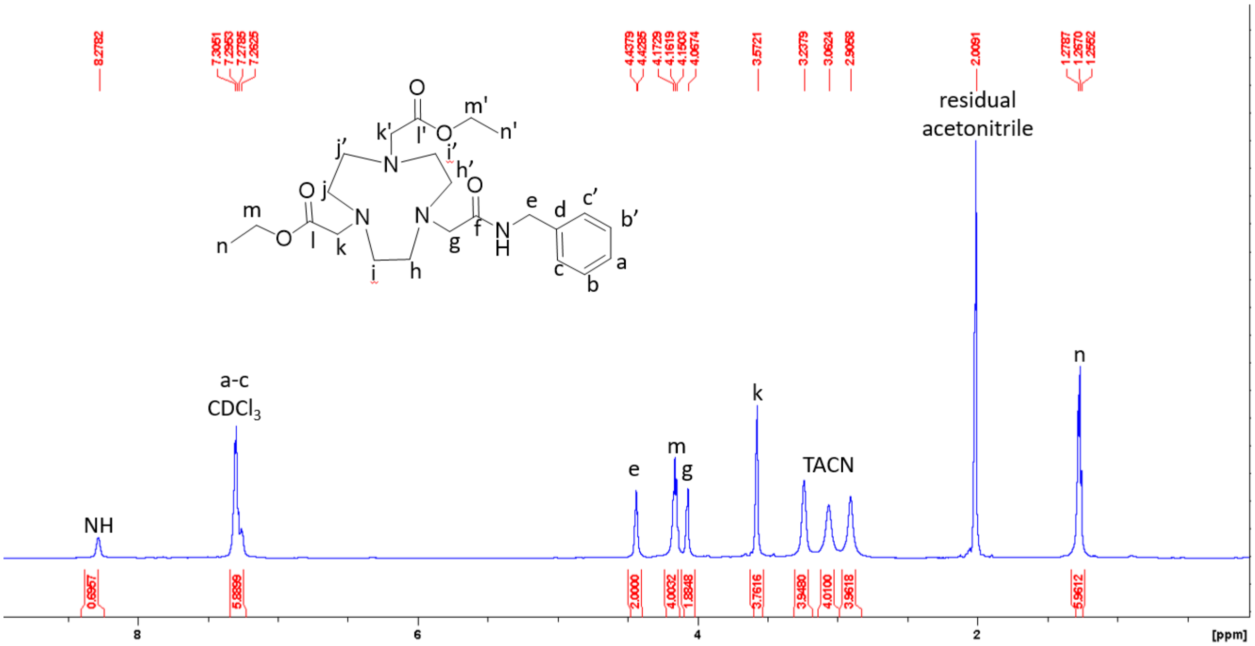

2.14 ^{13}C NMR **6** (600 MHz, $CDCl_3$)

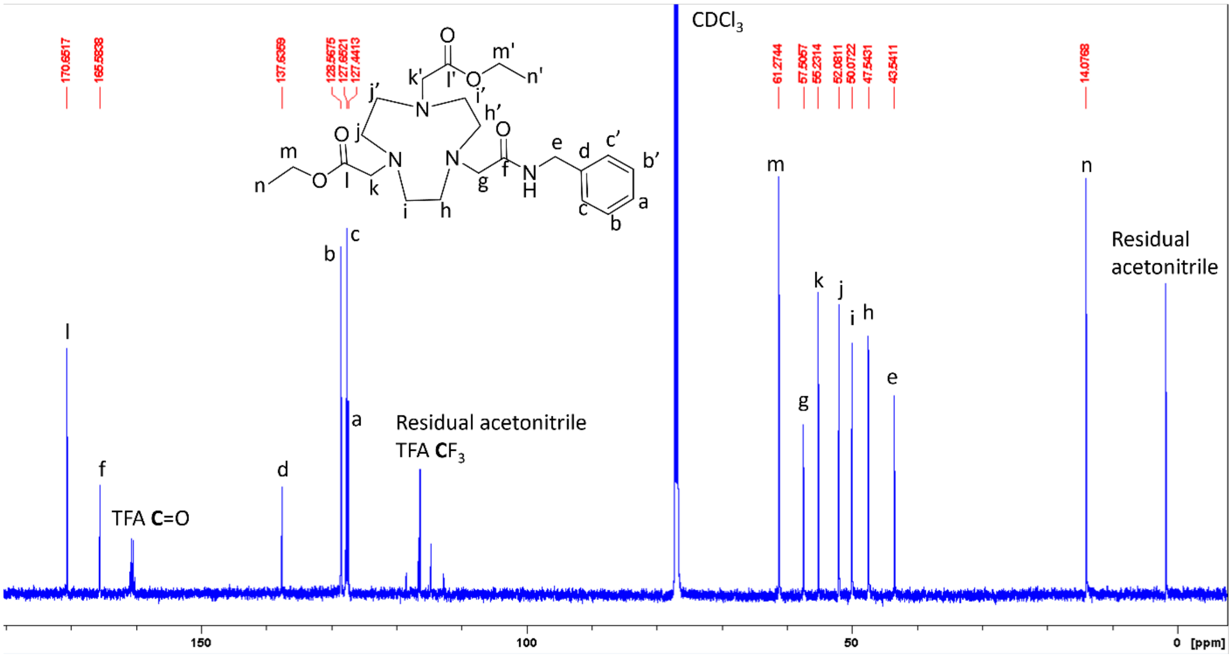

2.15 ^{1}H NMR **7** (600 MHz, MeOD)

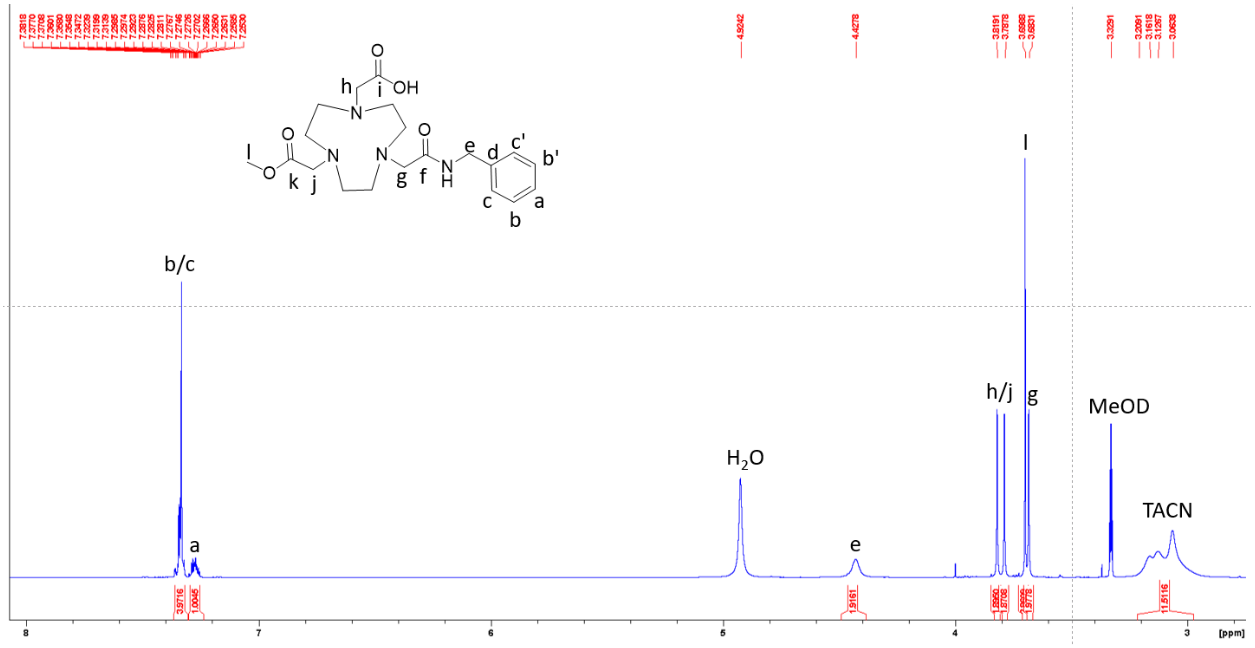

2.16 ^{13}C NMR **7** (600 MHz, MeOD)

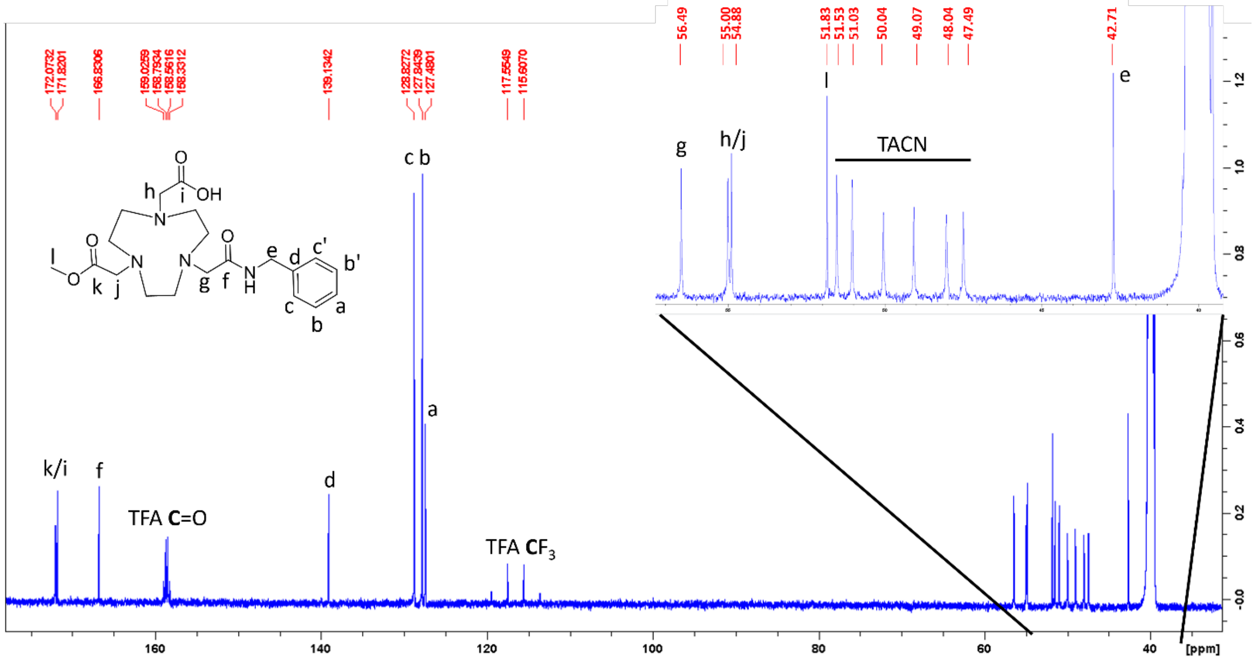

2.17 ^{1}H NMR **8** (600 MHz, $(CD_3)_2SO$)

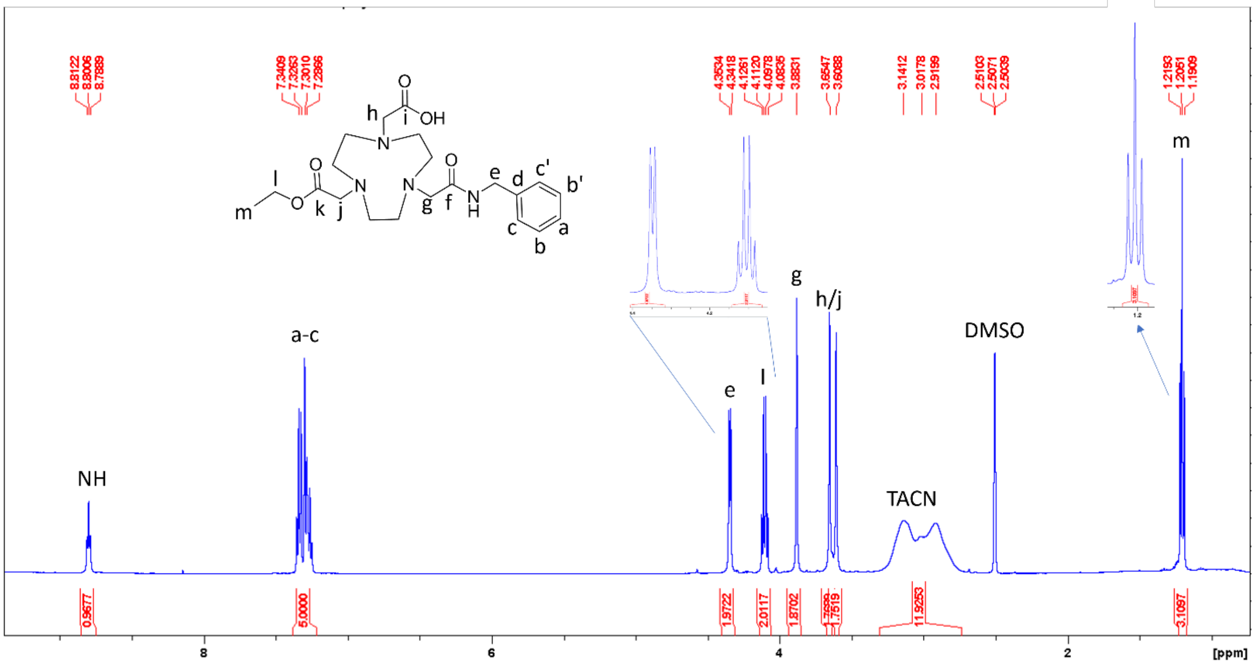

2.18 ^{13}C NMR **8** (600 MHz, $(CD_3)_2SO$)

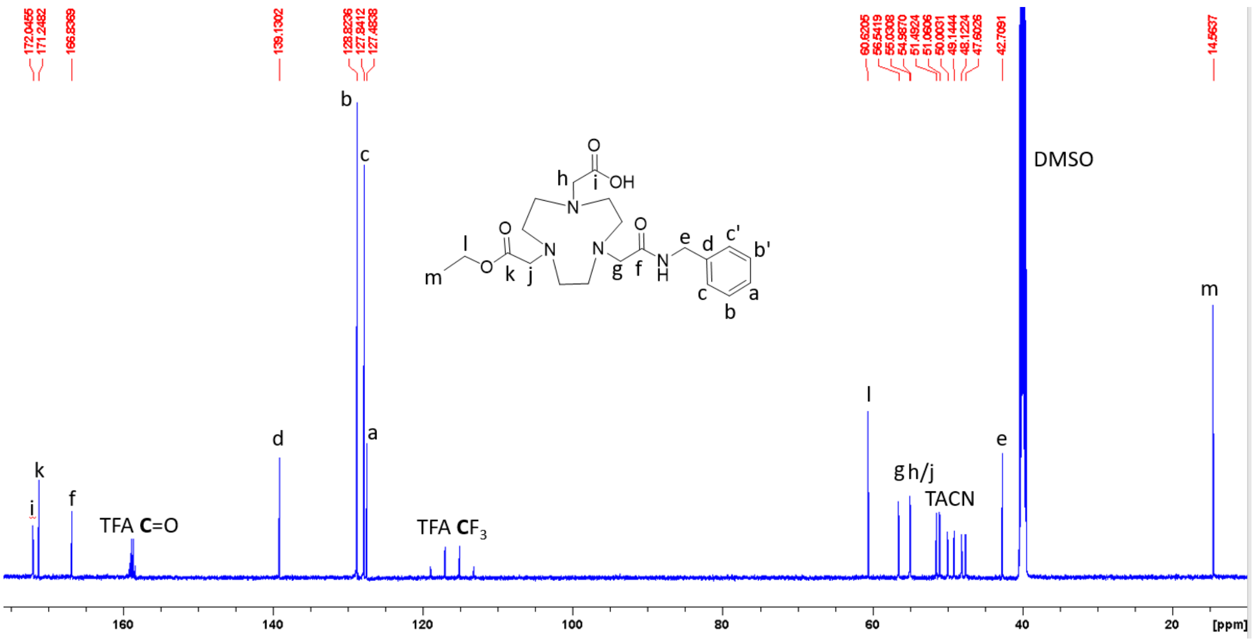

2.19 ^{1}H NMR **Re-3** (600 MHz, CD_3CN)

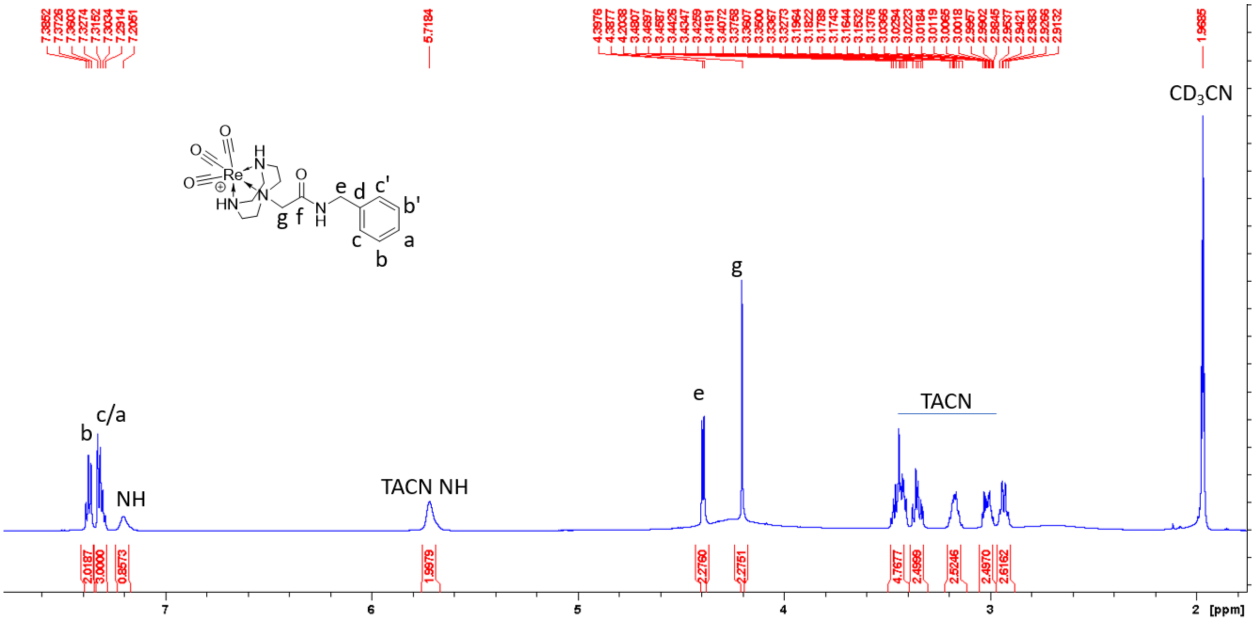

2.20 ^{13}C NMR **Re-3** (600 MHz, CD_3CN)

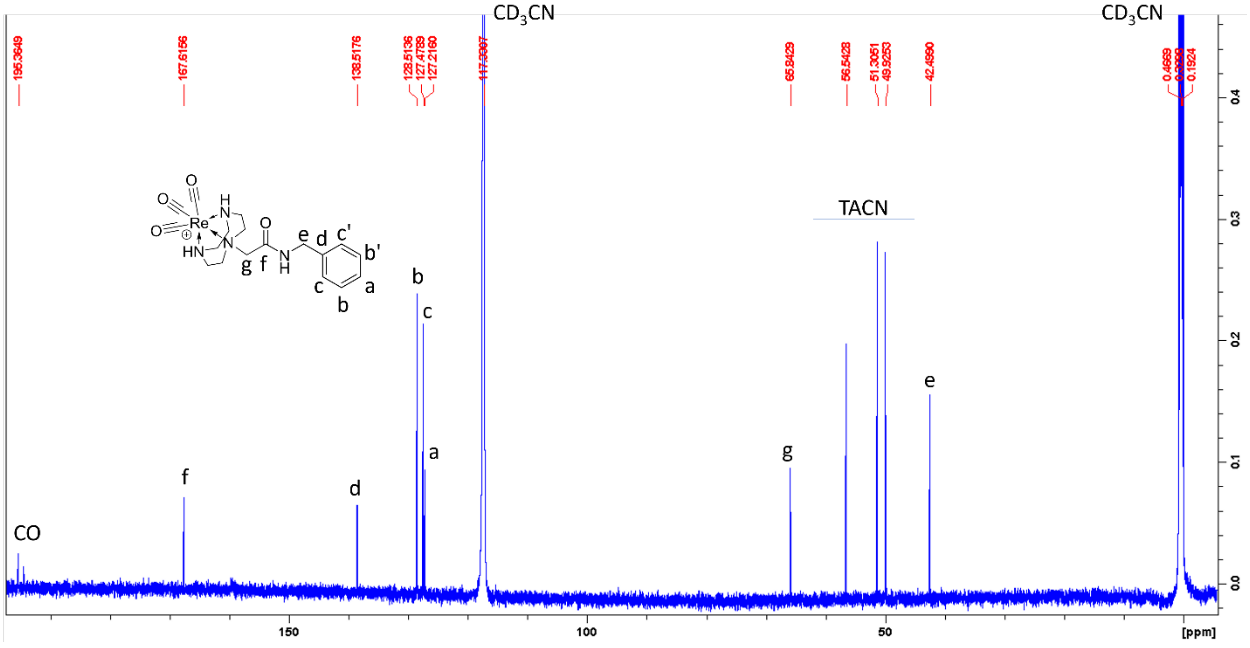

2.21 ^{1}H NMR **Re-4** (600 MHz, CD_3CN)

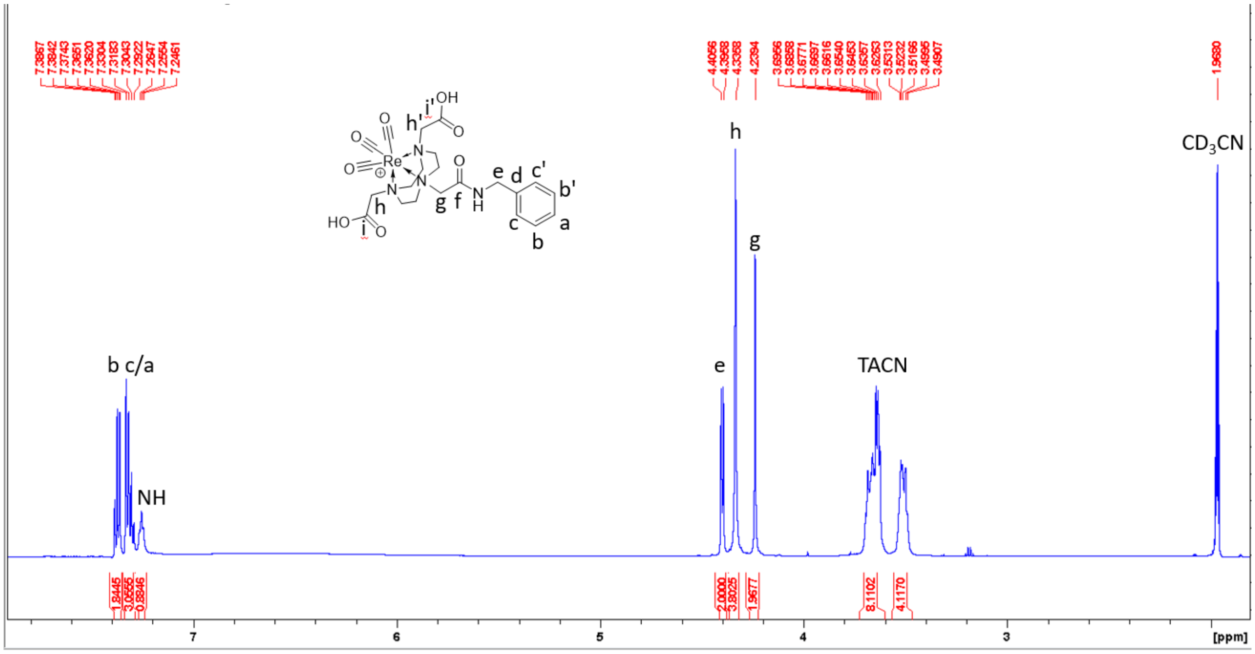

2.22 ^{13}C NMR **Re-4** (600 MHz, CD_3CN)

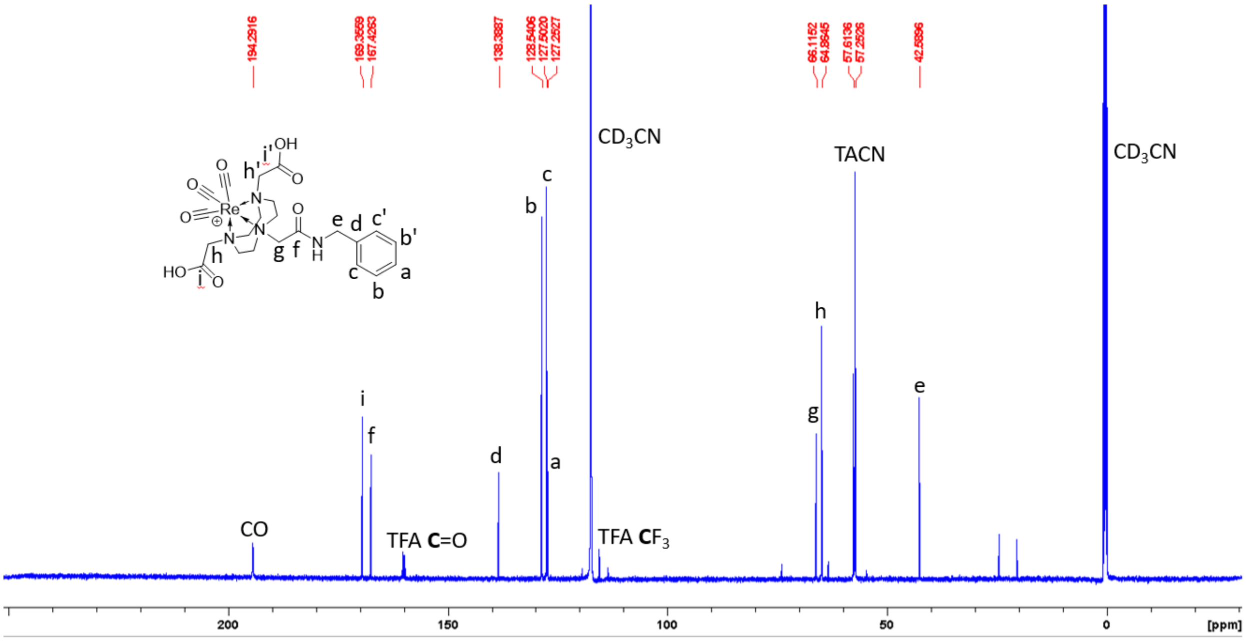

2.23 ^{1}H NMR **Re-5** (500 MHz, $(CD_3)_2CO$)

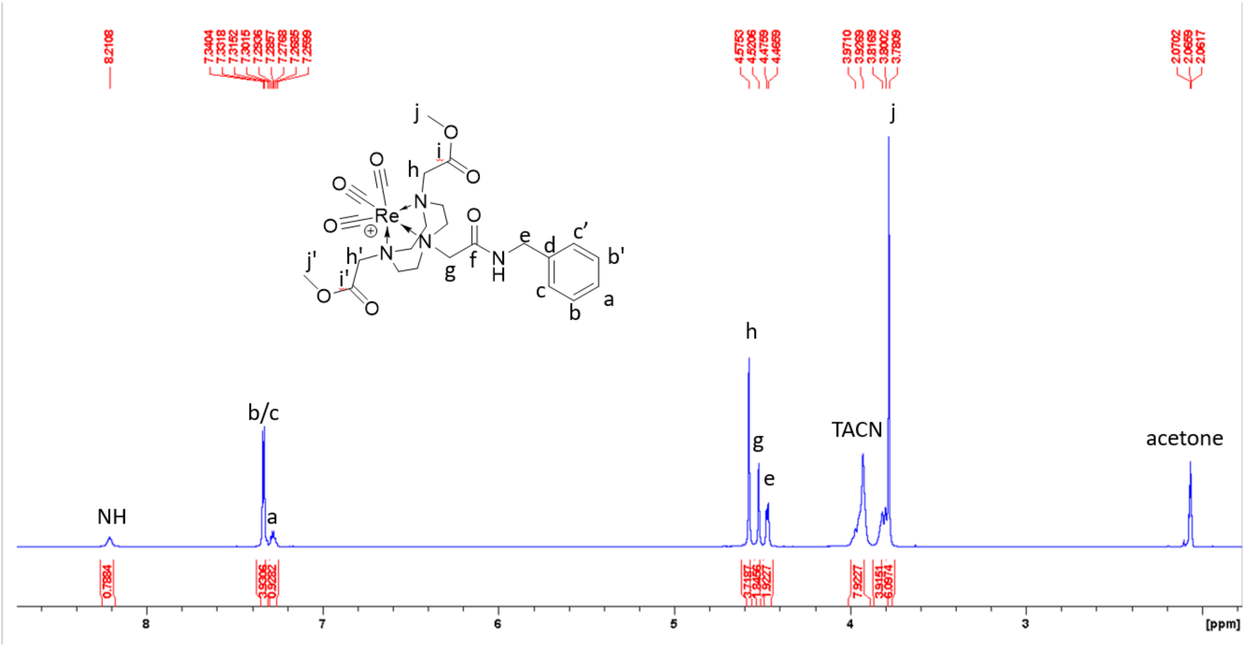

2.24 ^{13}C NMR **Re-5** (600 MHz, $(CD_3)_2CO$)

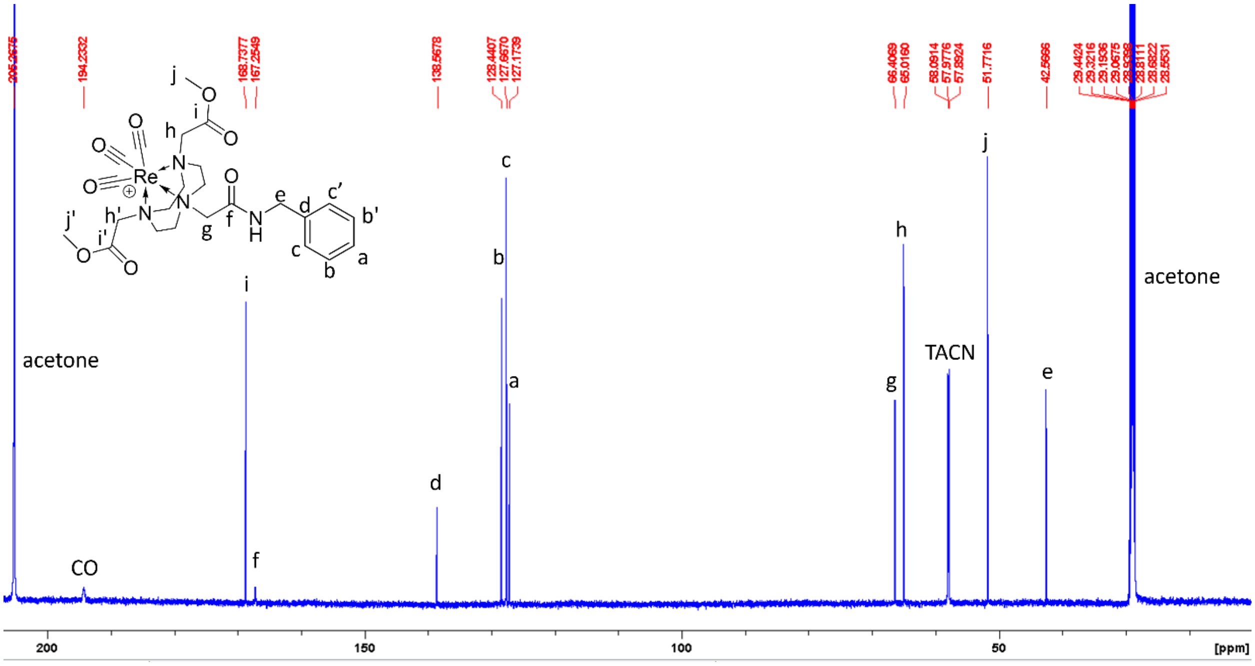

2.25 ^{1}H NMR **Re-6** (500 MHz, $(CD_3)_2CO$)

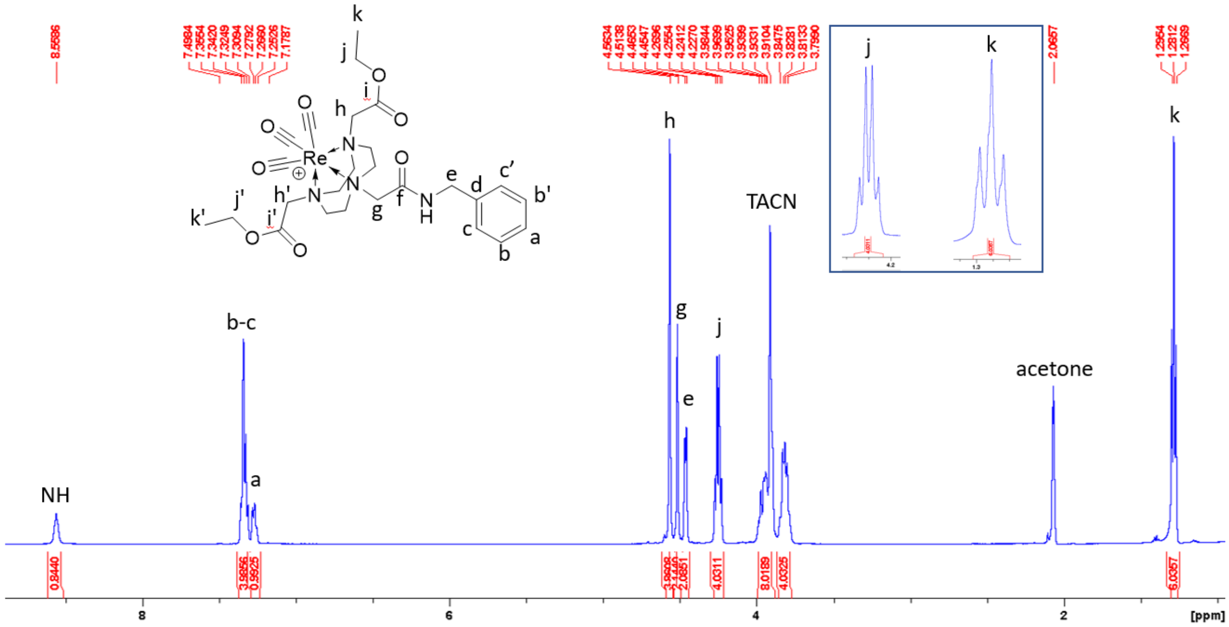

2.26 ^{13}C NMR **Re-6** (600 MHz, $(CD_3)_2CO$)

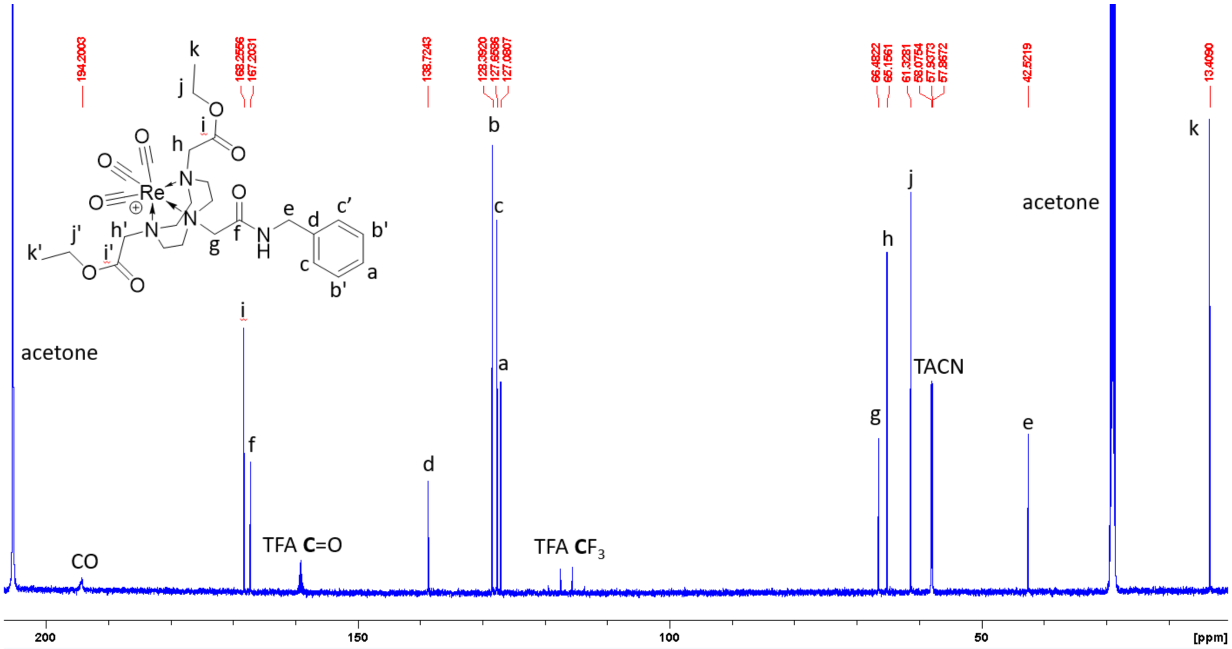

2.27 ^{1}H NMR **Re-7** (600 MHz, CD_3CN)

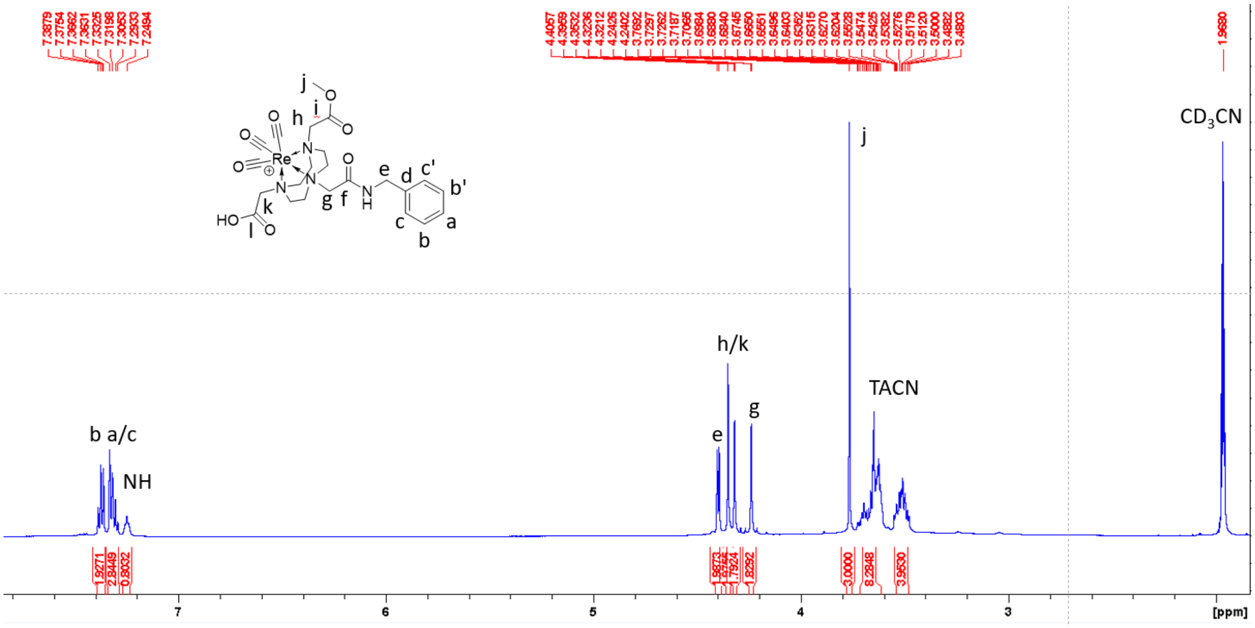

2.28 ^{13}C NMR **Re-7** (600 MHz, CD_3CN)

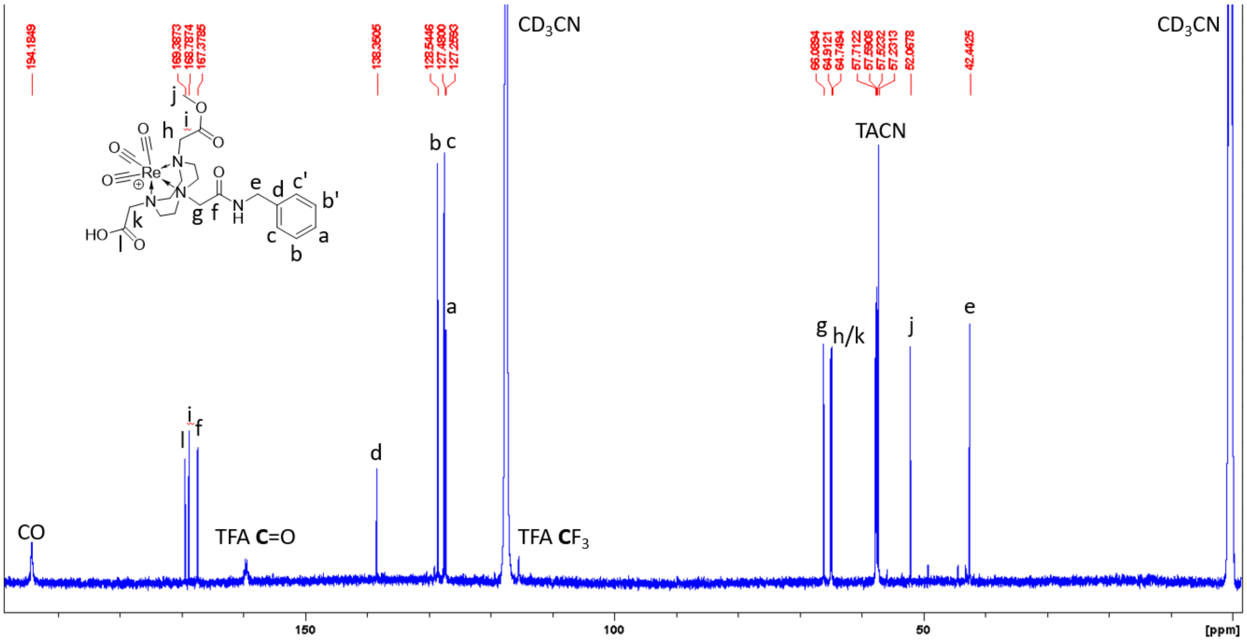

2.29 ^{1}H NMR **Re-8** (600 MHz, $(CD_3)_2CO$)

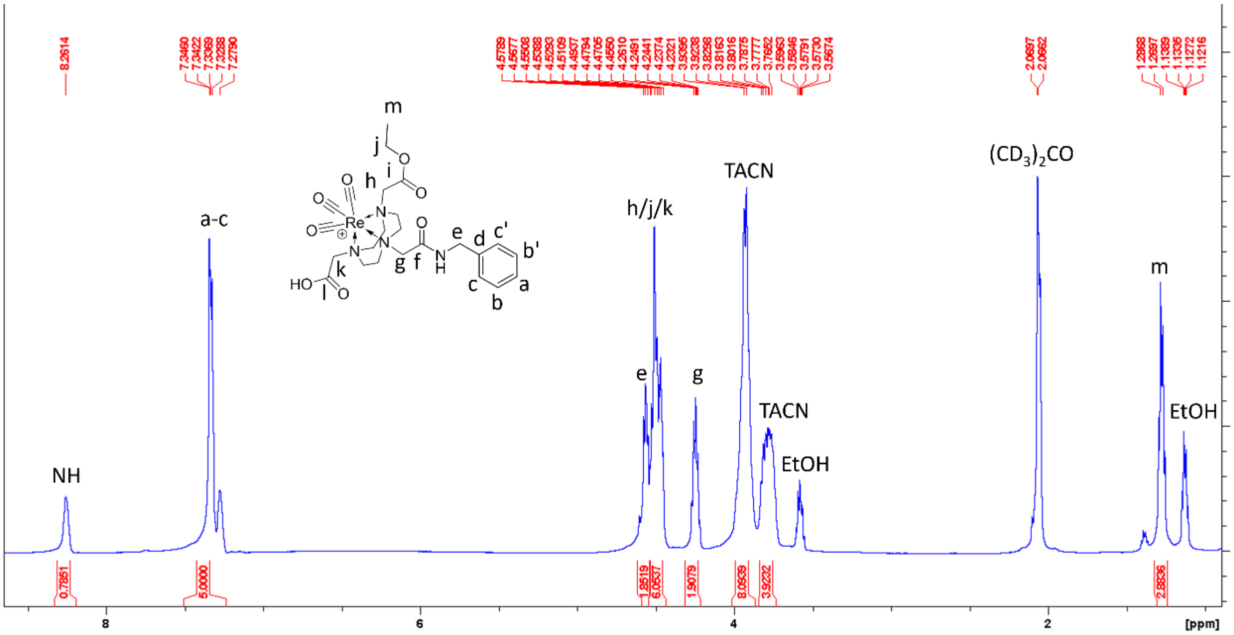

2.30 ^{13}C NMR **Re-8** (600 MHz, $(CD_3)_2CO$)

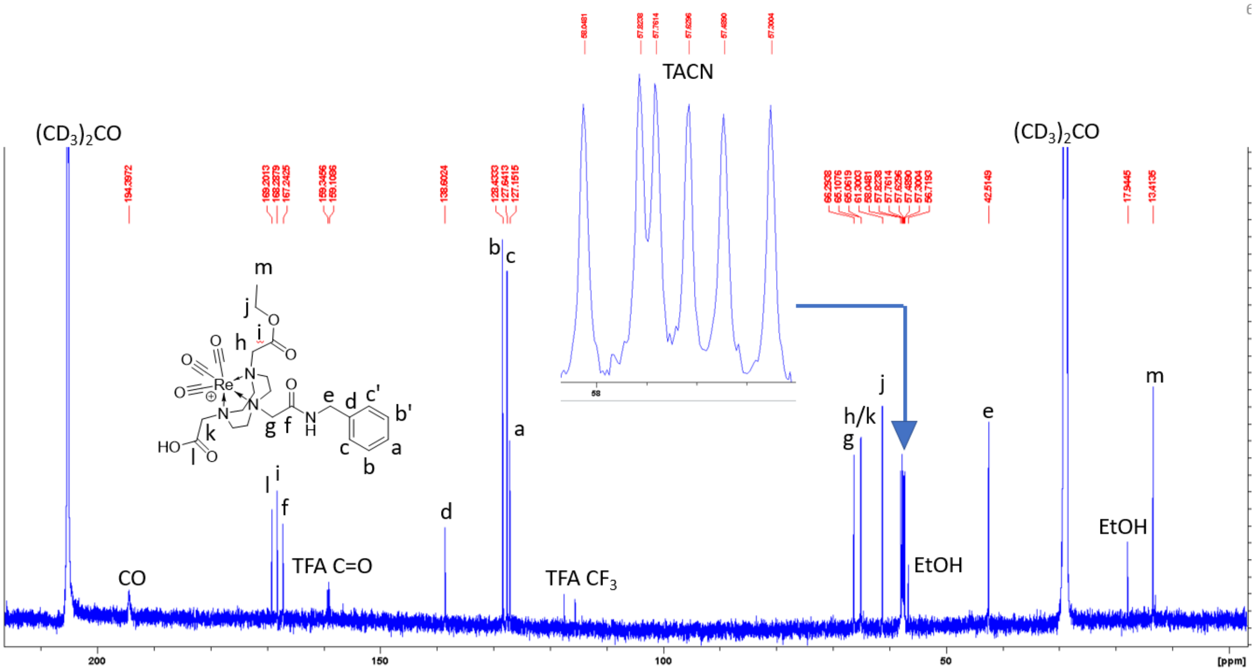

3. IR Spectra

IR spectra were taken on a Thermo Scientific Nicolet Summit Pro FTIR Spectrometer. Wavelengths between 500-4,000 cm^{-1} were recorded.

3.1 Re-3

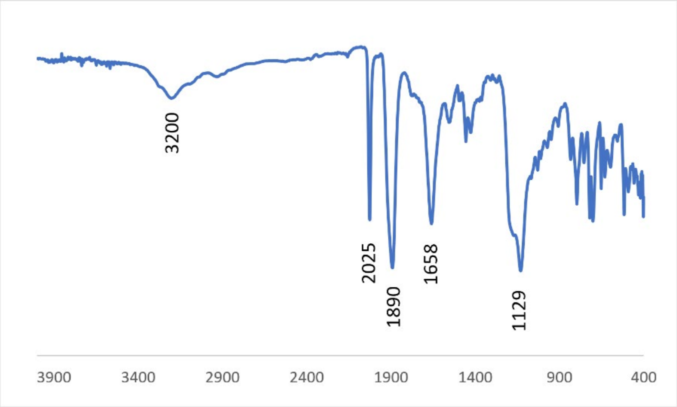

3.2 Re-4

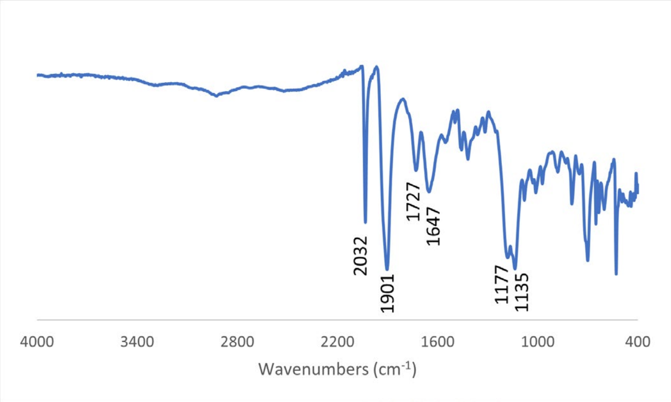

3.3 Re-5

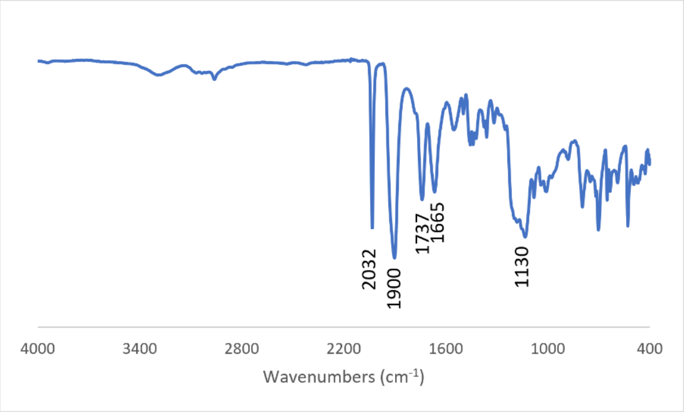

3.4 Re-6

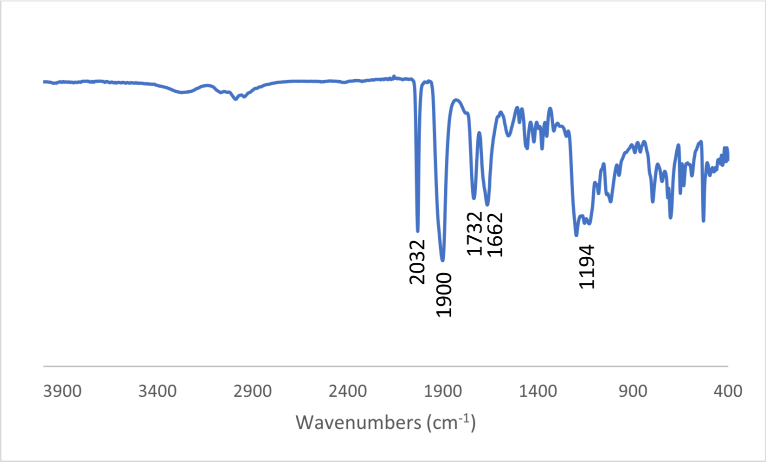

3.5 Re-7

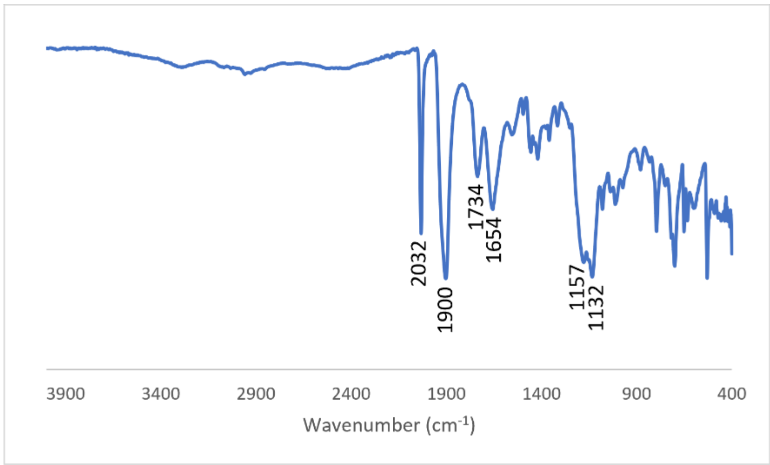

3.6 Re-8

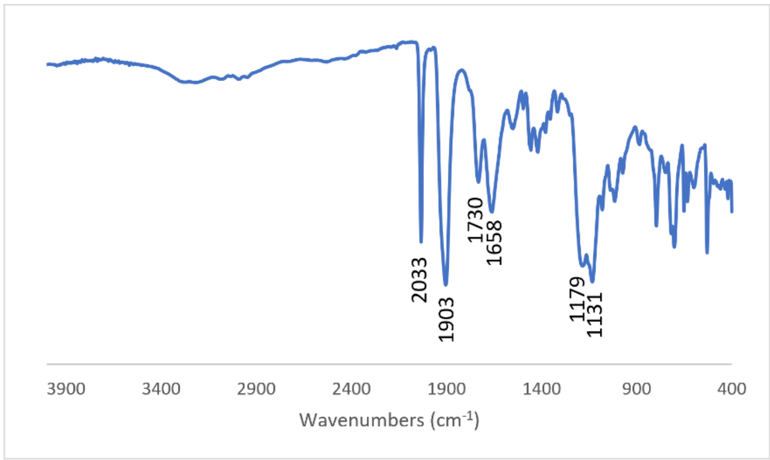

4. References:

(1) Braband, H.; Imstepf, S.; Benz, M.; Spingler, B.; Alberto, R. Combining bifunctional chelator with (3 + 2)-cycloaddition approaches: Synthesis of dual-function technetium complexes. *Inorg. Chem.* **2012**, *51* (7), 4051-4057. DOI: 10.1021/ic202212e.

(2) Blake, A. J.; Fallis, I. A.; Parsons, S.; Ross, S. A.; Schroder, M. Asymmetric functionalization of aza macrocycles. Syntheses, crystal structures and electrochemistry of $[Ni(Bz[9]aneN_3)_2][PF_6]_2$ and $[Pd(Bz[9]aneN_3)_2][PF_6]_2$.2MeCN ($Bz[9]aneN_3$ = 1-benzyl-1,4,7-triazacyclononane). *J. Chem. Soc., Dalton Trans.* **1996**, (4), 525-532.

(3) Alberto, R.; Egli, A.; Abram, U.; Hegetschweiler, K.; Gramlich, V.; Schubiger, P. A. Synthesis and reactivity of $[NEt_4]_2[ReBr_3(CO)_3]$. Formation and structural characterization of the clusters $[NEt_4][Re_3(\mu_3\text{-}OH)(\mu\text{-}OH)_3(CO)_9]$ and $[NEt_4][Re_2(\mu\text{-}OH)_3(CO)_6]$ by alkaline treatment. *J. Chem. Soc., Dalton Trans.* **1994**, (19), 2815-2820. DOI: 10.1039/DT9940002815.

(4) Schibli, R.; Schwarzbach, R.; Alberto, R.; Ortner, K.; Schmalle, H.; Dumas, C.; Egli, A.; Schubiger, P. A. Steps toward high specific activity labeling of biomolecules for therapeutic application: Preparation of precursor $[^{188}Re(H_2O)_3(CO)_3]^+$ and synthesis of tailor-made bifunctional ligand systems. *Bioconjugate Chem.* **2002**, *13* (4), 750-756. DOI: 10.1021/bc015568r.

(5) Makris, G.; Radford, L. L.; Kuchuk, M.; Gallazzi, F.; Jurisson, S. S.; Smith, C. J.; Hennkens, H. M. NOTA and NODAGA $[^{99m}Tc]$Tc- and $[^{186}Re]$Re-tricarbonyl complexes: Radiochemistry and first example of a $[^{99m}Tc]$Tc-NODAGA somatostatin receptor-targeting bioconjugate. *Bioconjugate Chem.* **2018**, *29* (12), 4040-4049. DOI: 10.1021/acs.bioconjchem.8b00670.

(6) Alberto, R.; Schibli, R.; Egli, A.; Schubiger, A. P.; Abram, U.; Kaden, T. A. A novel organometallic aqua complex of technetium for the labeling of biomolecules: Synthesis of $[^{99m}Tc(OH_2)_3(CO)_3]^+$ from $[^{99m}TcO_4]^-$ in aqueous solution and its reaction with a bifunctional ligand. *J. Am. Chem. Soc.* **1998**, *120* (31), 7987-7988. DOI: 10.1021/ja980745t.
